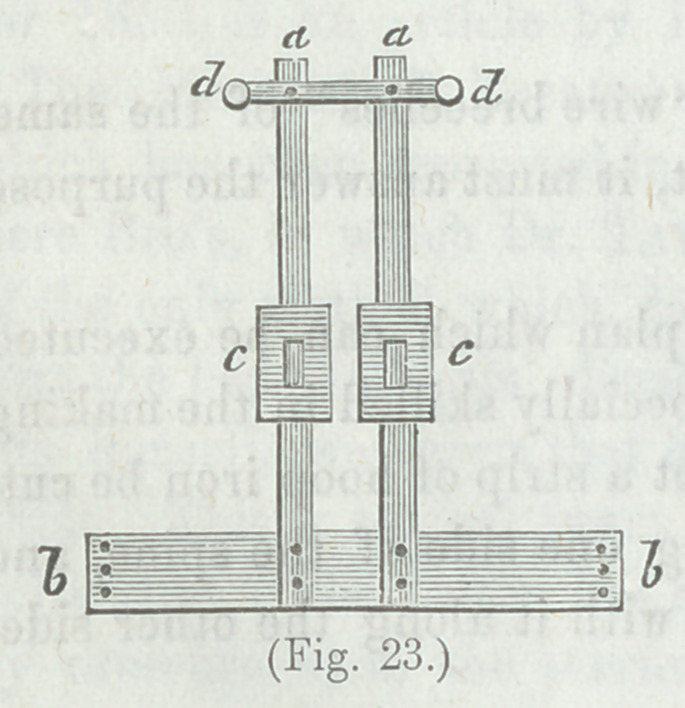# Report on Orthopedic Surgery, for the Annual Meeting of the Illinois State Medical Society, Held in Bloomington, May 2d, 1865

**Published:** 1865-08

**Authors:** David Prince

**Affiliations:** Jacksonville, Illinois


### PART SECOND.

### *Particular Diseases and Deformities.*

A. *Inflammation of the Joints of the Extremities and Resulting Deformities.*

First. HIP DISEASE—*Morbus Coxarius.*—The circumstances of inflammation of this joint single it out as the lion of its class.

It is the joint to which more force is applied than to any other joint in the body, with more varied and more extensive movements, with a closer contact of surfaces, and affording more extensive friction surfaces when the parts are inflamed. It is surrounded by stronger and more numerous muscles than any other joint, which, when spasmodically contracting, subject the head of the femur and the upper surface of the acetabulum to great pressure. On this account, synovial and cartilaginous inflammation, when once initiated, is more aggravated by movement and pressure, than in any other joint. The depth of the joint may be the reason for the obscurity of the sensations of discomfort in the early period of the disease, and for their reference to the parts lower down, and especially on the knee-joint.

The apparent elongation of the limb, from the tilting of the pelvis, may be a movement, in which the lumbar and ilio-lumbar muscles relax, to secure sympathetic relaxation of the ilio-femoral muscles, and thus diminish the pressure of the joint surfaces. After the period of muscular spasm has arrived, this is all reversed; the lumbar and ilio-lumbar muscles, contracting in their turn, from sympathy with the irritant contraction of the ilio-femoral muscles.

This primary dropping of the affected limb may be taken as a hint at the correct mechanical treatment in all stages. This branch of treatment, in the early period of the disease, has been sufficiently discussed; but in the periods of more aggravated suffering, its importance needs still more to be enforced; and this can best be done in connection with the farther notice to be taken of the pathology. As a most impressive presentation of the subject, attention is invited to the following quotations from *Barwell on the Joints, p.* 312:

"The nervo-muscular phenomena in hip-disease are so prominent and remarkable, that their evident results, as seen in the posture and apparent length of the limb, have chiefly attracted the attention of surgeons; and yet, the peculiar influence which they have upon the continuance of the malady, has escaped notice. Be it observed, that the constant and violent contraction does not simply produce ad- or ab-duction, according as one or the other set of actions may prevail; but as, from the direction of the muscles, it is evident, it must draw the thigh up and cause the head of the femur to press abnormally against the acetabulum. Thus, the pristine inflammation, having produced a contraction, the head of the thigh-bone begins to press with abnormal force and constancy in the upward direction. * * * To prove this position, we have only to look at a pathological museum. We shall find a few specimens in which the action is distributed over the whole joint surface; a very few indeed, in which the inflammation has chiefly attacked the lower portion or anterior part of the acetabulum and femur, but in a proportion so large as to render the above examples mere exceptions, the upper lip of the cotylon cavity and the corresponding portion of the head of the femur are ulcerating, while all the rest of the bone may be untouched. Such constancy of action can only be accounted for by the fact that abnormal muscular contraction produces pressure, and thereby ulceration of the parts. * * * Thus, the acetabulum is made to travel upward and also inward, whereby an opening into the cavity of the pelvis is not unfrequently produced. I say, that such evident yielding to pressure is not an exceptional case, but is the rule; that, when we find a hip-joint ulcerating in any other way and position, it is that some rare circumstance has caused a primary osteitis in that particular spot. It must also be remarked that, as the head of the femur travels upward, producing ulcerative absorption in that part, against which it so abnormally presses; it causes, beyond that point, an additional growth of bone, forming a new lip to the new cavity."

*Page* 317. "*Dislocation* of the head of the femur, from disease, or spontaneous dislocation as it is called, is an occurrence so unusual, that one is astonished at the general credence in its frequency. It is only about ten or fifteen years ago, that every hip-joint disease was supposed to end in this way; but, if a search be made in the College of Surgeons, St. Thomas', St. Bartholomew's, or others of our great pathological museums, there will be found very few specimens exhibiting, simultaneously, the signs of morbus coxarius and spontaneous dislocation.

On the other hand, it is by no means uncommon to find the head and neck of the femur shriveled to little more than a button-like projection, the acetabulum quite altered in form and place, and yet the bone retained in its cavity. * * * Spontaneous dislocation occurs only in cases of so cachectic a character that new bone is not produced beyond the focus of suppuration."

Second. *Inflammation of the Knee-Joint* appears in the same classes of tissues, in the same forms, with the same course and termination as the same disease of the hip-joint, only that the thinness of covering over the knee-joint renders the early diagnosis less obscure. There is less danger of being misled by the sympathetic pains which so often deceive in the early period of hip-disease. The indications for treatment are in all respects the same, and the facility of extension is greater. While the swelling and tenderness, on pressure, about the hip-joint sometimes renders it necessary in treatment to make counter-extension from the chest, this expedient is rarely necessary in disease of the knee-joint. The cases of *white swelling* of the knee, in which chest counter-extension will be most often found advantageous, are those in which extreme flexion has already occurred, with purulent burrowing tracks among the muscles of the thigh. By this mode of applying extension, the posterior part of the leg will be the only part of the affected limb subjected to pressure. This mode is equally applicable to flexion of the knee and of the hip-joints, and permits a considerable freedom of movement to the patient. With elastic fastenings, the muscles are permitted to contract and relax, securing to the joints a healthier nutrition than attends a uniform unyielding pull. At length, when the limb is brought out to some approach to a straight position, the counter-extension can be changed from the chest to the groin or the ischium, or to both combined.

Third. *Inflammation of other Joints.* Inflammation of the ankle, of the tarsal and phalangeal joints, hardly admit of extension, nor is deformity one of the frequent results, though stiffness may ensue. In the upper extremity, extension is practicable, but not so necessary, because the muscles are less strong, and there is no push put upon the bones, as is done in the lower extremities, and, besides, in walking, the weight of the limb amounts to a moderate extension.

Inflammation of a metacarpo-phalangeal joint may require extension, to avoid both stiffness and deformity, and this is easily effected by passing a rubber ribbon over the space between the thumb and the forefinger, and attaching each end to proximal end of a small splint, reaching from the lower end of the radius, to a point a little beyond the end of the affected finger, to which the distal end of the splint is attached by isinglass plaster. The slight, but constant pull, may save the finger from being rendered useless by stiffness or permanent flexion, or both combined, at the same time that the extension is a source of comfort to the patient.

The pioneer in the introduction of practicable forms of apparatus to answer the purpose of extension, is, doubtless, Dr. HENRY G. DAVIS, of New York. In a paper which he published in the *American Medical Monthly*, for May, 1856, referred to in his paper in the *Transactions American Medical Association*, 1863, he says, "There is one point in my mode of making extension, which I think, from the long experience I have had in its use, would be an improvement in the general mode, and it is equally applicable to all extensions and counter-extensions, those of fracture as well as contracted muscles, viz., the use of rubber as an extending power. This will act steadily and gradually, without any violence, and with very little suffering, in comparison with permanent fixtures. When contracted muscle is to be overcome, it steadily wearies it, until it quietly comes off conqueror."

We are permitted to copy the following cuts and descriptions of apparatus from Dr. Davis' report in *Trans. Am. Med. Association for* 1863:"—

"The apparatus, by means of which my treatment embodying the principles advocated in this paper can be carried into effect, is simple and easily explained.

"It must be borne in mind, that I have already said that the essential parts of the apparatus are, means of exerting an elastic, continually-extending force on one side, and a resisting counter-extending one on the other.

"The modifications it undergoes to adapt it to the various regions of the body, every physician can readily understand. I shall describe particularly the splint as applicable to the hip-joint. Reference to the wood-cut (Figure 3) annexed with further aid the reader.

(Fig. 3.)

"An elastic perineal band (*g* in the figure) really constitutes the extending, adhesive plaster strapping around the limb, concentering at a point a little above the external malleolus, the counter-extending power, while a metallic splint (*b*, *c*, *d*, *e*,) is stretched between these, and enables them to fulfil the indications proposed.

"The splint is composed of four parts, viz., an upper or pelvic portion (*a* in the figure), a thigh portion (*b*), a leg portion (*d*), and an ankle portion (*e*).

"The thigh portion consists of a metallic tube curved above (*b*), to correspond to the convexity of the thigh, and ending below in a short, straight piece, to which a long iron double-threaded screw (*c*), also hollow, is firmly secured. The leg portion is a straight metallic tube (*d*), closely investing the screw (*c*), and projecting but little beyond. It is attached to the ankle portion (*e*) in such a way that, in revolving it, in order to lengthen or shorten the instrument, only the screw investment with its nut is turned, while the ankle portion remains unmoved. This ankle portion (*e*) consists of a triangular-shaped, flat piece of metal, covered by a buckle (as seen in the figure).

"The pelvis portion (*a*) is more complicated than the others. A slightly curved strip of steel half an inch or more wide, from four to six inches (both the length and width varying according to the size of the whole splint) long, is rivited through its centre to the free extremity of the thigh portion, and admits of rotary motion. At one end of this strip of steel a buckle, and at the other the perineal band is attached, and the whole of it is well cushioned. The perineal band is formed of two bands (*f* and *g*), of a length, width, and strength varying according to the size of the apparatus and the circumstances of its application. One (*f*) is longer than the other and inelastic, being made entirely of strong cotton or linen webbing, the other (*g*) is, as it were, an oblong bag of India-rubber webbing (formed by sewing two stripes of rubber webbing together) filled with saw-dust, obtained by sawing across the fibre of pine wood (not lengthwise), tipped at each end with some of the inelastic webing (such as (*f*) is made of). While the inside elastic band keeps up the extension required, the inelastic sustains any weight that exceeds the extending force as then applied to the patient. It is this arrangement that enables the weight of the body to be borne without harm, as in walking, and that prevents injury from excessive weight or pressure upon the articulating surfaces in cases of accident. Thus, for instance, the head of the femur would, in walking, be violently thrust upward, as the elastic band would yield to an increased weight, were there no inelastic, unyielding band to prevent it; yet, it is obvious that this inelastic band does not interfere with the predetermined amount of tension to be exerted by the elastic one. (This amount of extension is determined and regulated as follows: Buckle the two bands unequally, *i.e.*, let the loop formed by the outside band be longer than that of the inside, and attach a weight to the latter. The number of pounds requisite to stretch the one loop to the exact length of the other represents the amount of extending force the instrument will exert, when exactly thus buckled, when applied upon the limb.

"I will add that here the amount of extending force should be ascertained in every instance before fastening the splint upon the patient; this amount is not to be varied by altering, by means of the screw, the length of the instrument, but by adjusting the two bands.)

"I have found the rubber webbing, with napkin protection to the skin, to be much superior to the rubber tubing. The tubing is seldom of the right elasticity, and is more liable to to heat and excoriate the skin than the webbing, for the reason that it does not absorb the secretions, nor allow the air to come in contact with the skin.

"The granular stuffing moves easily on itself and displaces readily, so as to equalize the pressure, as, for instance, over the adductor tendons in the groin.

"The long splint is best adapted to the majority of cases. Some years ago, I was in the habit of applying a shorter one (Fig. 4) to the femur alone. This leaves the knee at liberty, and in so far is an accommodation to the patient, but otherwise is not so effectual.

"I also generally use the long splint for disease of the knee-joint, applying the adhesive plasters only to the tibial portion of the limb. I have devised, however, a very convenient instrument

(Fig. 4.)

(Fig. 5.)

(Figs. 5 and 6) for extension at the knee-joint, that admits of flexion and extension of that joint, which in cases not too severe is sufficiently effectual.

"Extension should be constant; when not accomplished by the splint, it should be by means of a weight and pulley.

(Fig. 6.)

(Fig. 7,)

(Fig, 8.)

"*Mode of Application.*—Cut from a piece of adheisve plaster, spread on twilled goods and kept until the oil entering into its composition has become oxidized, two strips from $1\frac{1}{4}$ to $1\frac{1}{2}$ inches wide, of the length of the limb from the pubis to the malleolus, and two strips a little narrower in proportion to the others, but one and a-half times as long. Fold about an inch and a-half of one extremity of each of the first cut strips upon itself, the adherent sides to each other, and apply one on the outside and one on the inside of the limb, commencing with the folded end about two inches above the outer and inner malleoli, and extending it up in a straight line.

"The other two strips are applied spirally around the limb as follows; Commence on the lower or folded extremity of the straight strip above the outer malleolus, and wind around in front and back, so that the two spiral strips meet in front, a little distance above the patella (as very well depicted in the woodcut, Fig. 7).

"Next, sew a piece of firm, *inelastic* (linen or cotton) webbing, about 1¼ inches wide from six to eight inches long, to the lower extremity of each straight strip, taking particular care to include in the attachment the ends of both spiral strips above the external malleolus. The limb is then closely and firmly enveloped with a common roller bandage, from the foot upwards (as shown in Fig 8), the pieces of webbing only being left outside free. Now buckle the ankle portion of the splint upon the external face of the limb by means of the webbing; protect the skin of the groin and parts to be covered by the perineal band by a piece of old, soft napkin or table linen, several times folded and secured by a few stitches; and having previously adjusted the two bands composing the perineal band, as mentioned on *p.* 163, fasten the latter around the thigh, always taking care to have the buckle on the pelvic portion of the splint in front; the screw of the splint regulates its length, so that the required amount of extension can be secured. When all is correctly arranged, and proper extension made, the upper extremity of the splint should fall just below the crest of the ileum."

(Fig. 9.)

Figs. (9 and 10) are further modifications of extending apparatus, and (Fig. 11) illustrates the manner of connecting and fastening the two portions of the extending shaft.*

DR. LEWIS A. SAYRE, of New York, has modified DR. DAVIS' splint chiefly, by making the extending shaft, in great part, of wood,

* We learn from Dr. Davis that he is preparing to publish all his improvements in surgery in one volume, in order to give the profession the benefit of his methods of treatment, which are the result of many years experience.

which slides in a metallic trough, and fastens by a catch falling between the teeth of a ratch. The counter-extension, as in the apparatus of DAVIS, is the usual oblique band over the groin and perineum.

(Fig.10.)

(Fig. 11.)

Mr. RICHARD BARWELL, of London, has, also, a less ingenious modification, figured in his work, *On the Diseases of Joints*, and also in his little book on *Club-Foot, &c.* He claims originality for this, but it is said that one of DAVIS' machines was exhibited in some of the London hospitals before that of BARWELL was invented.

Dr. EDMUND ANDREWS, of Chicago, has devised an extending apparatus, in which the extending shaft is made of gaspipe, with a screw extension, as in the apparatus of DAVIS, but Dr. ANDREWS places the splint upon the inside of the limb, with a sort of crutch head, fitted to the shape of the coverings of the pubis and ischium, to receive the weight of the body in walking, and to be, at all times, the medium of counter-extension, or, more properly, counter-pressure.

All these forms of extending apparatus require more skill in constructing them than the general practitioner can ordinarily find in his nearest blacksmith and carpenter, and I have endeavored to perfect a plan of apparatus which will enable all practitioners, both in town and country, to give their poorest patients the benefits of treatment which, for practical results, may be equal to that of experts in this particular branch.

After trials of various forms, I have settled down upon the plan illustrated in Figure 12. The shaft is placed upon the

(Fig. 12.)*

outside of the limb, and a collar is adapted to the thigh, to press like a crutch upon the ischium. The whole splint (adapted to the left side) is seen in an oblique aspect. The shaft is of hard wood, and has the collar for the ischium upon the upper end, (lower, as seen in the cut,) and upon the other end, an iron foot rod, which is intended to pass through the heel of the shoe. The shaft is made so long that the weight of the body cannot come upon the foot. The collar, which can be made of hoop-iron by any blacksmith, is about 13-16 of a circle, 8-16 behind, and 5-16 before, and should be carefully padded.

For children, this should be temporary, and made of cotton and muslin, in order that it can be easily and cheaply renewed as often as it becomes wet.

The small figure is an end or profile view of the iron collar attached to the end of the wooden shaft.

The shaft may be made extensible and adjustable, but this requires the skill of a professional instrument maker, and is expensive, while, if made for a particular individual, there is no advantage in it.

The collar should be beveled behind, to apply to the ischium without discomfort, but in front this is not necessary. The open space comes opposite the great vessels descending from the groin. The collar is such a segment of a circle as not to need a strap to hold it in position.

The readiest and most efficient expedient for extension, at the same time that no inconvenient projection is necessarily made beyond the foot, is that invented by BARWELL, and figured in his little book, *The Cure of Club-Foot.* It consists in attaching a parallelogram of tin to the leg, by means of adhesive plaster, so as to get the point of attachment of the extending agent near the knee-joint. A spring of elastic rubber or a spiral steel spring can then be attached above, to the upper end of this piece of tin, and below, to the lower end of the extending shaft, which may terminate either above or below the foot. The accompanying figure illustrates the mode of applying the tin and plaster.

(Fig. 13.)

Figure 13. Barwell's method of attaching a piece of tin to the leg.

*d* The lower end of a strip of plaster attached to the leg, and upon the outside of which, the tin *e* is applied. The plaster is then turned up against the tin, and circular strips of plaster are applied, covered by a roller.

*f* A loop for the attachment of the extending spring.

By means of this expedient and the ischiatic crutch, extension upon the hip-joint and the knee-joint can be kept up while the patient walks about, the whole weight of the body coming upon the shaft of the crutch, and being received by the ischium. The muscles passing the hip-joint direct the movements of the limb, but impose no pressure upon the hip-joint, their own contraction being counteracted by the spring which makes the extension.

It is convenient to attach a pretty long heel to the shoe, with a hole in it just large enough to receive the angular spike upon the lower end of the shaft. As this passes through the heel of the shoe, any pull upon the shoe expends its force upon the shaft, through which it finally comes upon the ischium. When the pressure upon the ischium becomes in any degree uncomfortable, the patient puts his hand upon the upper end of the shaft and pushes it down, and relieves the ischium from all pressure whatever. As the rubber springs yield, the extension is increased, at the same time that the pressure upon the ischium is relieved.

At night, the shoe can be taken off, and the elastic extending straps can then be attached to the angular spike, in order to keep up the extension while the patient sleeps.

B. *Lateral Curvatures of the Spine—Skoliosis.*—Deviations of the spinal column to the right or left of the straight median line, or in one part to one side and in another part to the other side, must depend upon deficiency in the bones, the ligaments and cartilages, the muscles, or in some necessity of the spine to deviate from the straight line to tilt the pelvis toward a short limb, or to conform to changes in the volume of the organs within the chest, or upon several of these causes combined.

The classification of these curvatures, in accordance with their pathological causes, must often be very much in doubt, because the opportunities for *post mortem* examinations in persons dying from other diseases, during the early periods of lateral curvature, must be rare, and, when they do occur, the lesions may be so difficult to distinguish, or the primary alterations in one set of organs, and the secondary changes in another set of organs, may be so equal, that the distinction of cause from effect may be impossible.

In the present state of the science, the classification must be made more upon general considerations, than upon observed pathological states of the organs.

I. The simplest division is based upon supposed weakness of bones in proportion to the muscles rapidly developed.

II. Disease of the ligaments, and bones weakening them, not only relatively, but absolutely.

III. Fatigue of overtaxed, feeble muscles, shirking their work upon the ligaments.

IV. Spasmodic contraction of the muscles of one side affecting a single muscle, or a greater or less number, from irritation in the brain, the spinal cord, or in the course of the nerves, or reflected from some place of irritation outside of the central nervous system.

1. A person, during the growing period, has a course of fever, and upon recovering, gains two or three inches in stature in a very short time, and acquires a curvature while the muscles are in active and healthful exercise.

This picture is in obvious analogy with cases of curvature of the bones of the lower extremeties of infants, in whom the muscles develop more rapidly than the bones, or in whom the ambition of parents or nurses leads to the too early teaching of the child to stand and walk. If the tibiæ were divided into segments, with intervening cartilaginous and ligamentous material, like the arrangement of the spine, it would, doubtless, bend; the bony substances, yielding as readily in the multiplied pieces as in the single piece.

The muscles, outrunning the bones in development in both cases, the principles of treatment must be the same in both. The chief indication must be, to place less weight, or for shorter periods, upon the bones.

For cases of this class, sufficient rest in the horizontal posture is more important to be enjoined than any system of movements.

The movements which are practiced, however, should be so diversified, as to bend the spine in every direction, of which it is capable, and so amusing as to be a pastime to the patient. Of all practices, however, sitting on a bench at school, is most injurious to a person of this class. Permanent distortions, from this class of causes, may require apparatus, as a bow-leg requires a splint and persistent force to straighten it.

2. A young person, falling from a height, or from some other form of violence, sprains the ligamentous fastenings of the spine, and, after several months, a curvature, chiefly on one side of the median line, is noticed, of such a marked character as to seem to have occurred suddenly, while very little complaint is heard from the patient, whose lassitude, and indisposition to the sports of youth, are out of proportion to any feelings of pain or discomfort arising from the disease.

This picture is in plain analogy with those cases of white swelling of other joints, which originate in the ligamentous, synovial, and cartilagenous investments of the bones, and which are very slightly painful, on account of the paucity or absence of nerves of sensation in the tissues affected, or by their failure to be awoke into painful activity by the low grade of the existing inflammation; producing changes of volume so slowly as to give the nervous filaments time to accommodate themselves to the changing relations.

It may be assumed that, when tissues affected with acute or sub-acute inflammation, pass into a state of chronic inflammation at the ordinary period of the termination of the acute disease, there must be some continued causes of irritation, or some cachexia, original or acquired, temporary or permanent, which causes the continuance of the disease in the chronic form. In this sense, there may be a constitutional disease, requiring constitutional treatment, as well as rest, of the parts affected. The cases in this class differ from those of the first class, in which there is no disease; only disproportionate development.

It is obvious that relief of the inflamed ligaments, from the strain of the weight and movements of the body, is of the first and highest importance.

Nothing can do this so effectually as the horizontal posture; but if the deformity has become constitutional, and resists the attempt to strengthen the spine by the hands of the surgeon, the curvature will never straighten itself. Force, from without, must be persistently applied by suitable apparatus.

3. A growing person, generally a girl, of lax joints and slender muscles, is restrained from the diversified movements which are so delightful to children and youth, while the desire of securing a straight spine, and slim waist, leads to the application of stays, which interfere with the free play and rocking of the vertebræ upon each other, while, from the partial disuse of the lateral muscles of the spine, they acquire a more marked degree of atrophy than the other muscles of the body; while yet a monotonous sitting posture, under restraint at school, presents the strongest temptation to rest the muscles, by permitting the weight of the head and shoulders to curve the spine, as far as the embracing stays and the ligamentous connections will permit, which, by habit, will come, at length, to be always on one side in one part of the spine, and on the other side in another part.

This picture describes the muscular class of curvatures — of artificial production, caused or aggravated by the means employed to prevent or cure them.

A girl, left to choose her own amusements and occupations, never would acquire this kind of curvature. The instinct of movement would lead to diversified action of the muscles, and, on becoming fatigued, she would lie down. Rest, in the horizontal posture, would be always more grateful and more complete, than that rest of the muscles, which is secured by allowing the weight of the parts alone, to come upon the ligaments, and the surrounding artificial supports.

As far as a person can bend the spine, by a moderate effort, so far it will deviate in this kind of resting; and, coming to be habitual in one direction for each portion of the spine, the ligaments will at length elongate on one side, and shorten on the other; as in the production of deformity of other joints, until a habitual and fixed curvature is the result.

This class differs from the other two, in being the result of the vicious tastes and usages of fashionable society, or of the excessive regard for intellectual training, in disregard of the necessities of the physical constitution.

I am constrained to quote, on this subject, Dr. Beal, to show how well this it was understood, thirty or forty years ago:

"Inactivity is not alone sufficient to account for that degree of muscular debility which induces spinal deformity. In warm climates, women take less exercise than they do in this country (England), and yet, curvature of the spine is infinitely more rare in such countries than in our own. In hot climates, all people indulge more in the recumbent position; reposing themselves, when fatigued, as Nature dictates. With us, a girl, from the age of ten, is obliged, throughout the day, to maintain a constrained position of the body. She is not permitted to rest the muscles of the back, however weary, and the admonitions of parents and tutors are unceasing, to keep herself erect. In this way, the muscles of the back are overstrained. * * * The comparative immunity of females of the higher classes in hot climates from spinal distortions, may, in part, depend upon their freedom from the pressure of stays and bandages. Mr. SHAW has some good observations on this subject:—'It is, perhaps, correct to say, that the less exercise a child takes, the more does she require general muscular relaxation in the recumbent position; and, that the lighter and more sedentary the pursuits are, the more necessity there will be either for active exercise or general relaxation. Thus, in warm climates, where active exercises cannot be taken, the due relation of parts, or balance of the system, is preserved by great indulgence in the recumbent position.' "

On the theory of exclusive muscular action, in maintaining the erect posture, a soldierly attitude should be the one which all persons would choose while standing, for the more nearly perpendicular the spine is kept, the more easily must the weight of the head and trunk be balanced. Yet this is contrary to all experience, for soldiers themselves, as soon as they are released from discipline, immediately assume attitudes in which the spine makes first one set of ligaments tense, and then the other. It is not so much to rest the muscles of the legs, that the weight of the body is thrown first upon one and then upon the other, as to rest the muscles of the spine, by throwing the burden of balancing upon the ligaments.

From all this, the indication is plain, to give the muscles variety of exercise, with frequent and abundant rest in the horizontal posture, saving the ligaments from any persistent strain, and, in confirmed cases, to strain the ligaments in the opposite direction, till they acquire equality of development on the two sides.

It used to be advised, in this class of cases, to carry a bag of sand, or other considerable weight, upon the head, to tempt the muscles to hold the spine erect, as the easiest way of sustaining the burden. If the muscles fail to appreciate this advantage, in sustaining the weight of the head and shoulders alone, it is difficult to see how they should become more shrewd, when the weight of a bag of sand is added. The truth, probably, is, that in both cases, the muscles, as soon as they are fatigued, attempt to shirk the burden, by allowing the spine to settle to the extent of its lateral flexibility, throwing the strain upon the ligaments. Thus, the greater the weight, the greater must be the strain, and the greater the consequent yielding and gradual aggravation of the curvature.

The system of movements devised by Dr. LING, of Sweden, and, with more or less modification, taught by DIO LEWIS, and others, are well suited to prevent this class of deformities, and to correct them in their early stages. It is a happy reform, to introduce them into schools as a part of the regular daily exercises, not only for the purpose here considered, but to secure better general muscular growth, better digestion, and more balanced developments.

In unconfirmed curvature, from muscular weakness, an important means of giving vigor to the muscles, is friction upon the skin over them. The efficacy of this, in imparting muscular tone, is well enough understood by every groom, and it is a pity that it should be so much neglected in human hygiene. Once, or oftener, in the twenty-four hours, the patient should lie upon her face, with the whole length of the spine exposed, when passes should be made slowly, and with a considerable degree of pressure, from the occiput and sacrum, the passes all being toward the sacrum, and continued fifteen minutes at each period. It is convenient to practice this friction just before bedtime, for its additional effect in procuring good sleep. To give greater adhesiveness to the surface of the hand, it may be moistened with some alcoholic preparation.

4. Lateral curvature, from spasmodic action of muscles, finds its best illustration in torticollis, or wryneck, in cases of which, rigidity of the sterno-cleido-mastoid muscles is associated with spasmodic action of spinal muscles of the same side. Fortunately, the cases of curvature of this class are few, though the twisting of the neck and tilting of the head, from the action of the sterno-cleido-mastoid, are common enough.

These cases usually arise, like strabismus, from sympathetic disturbances of nervous function, and the most successful treatment must be the early removal of the disturbing causes. The permanency of the deformity depends upon the hypertrophied and shortened condition of the muscles, after the irritating disturbances have passed away, or upon the same ligamentous condition of contracture.

Where these irritations have a hysterical character, the effects may be expected to be more transient, while contractions resulting from the reflexion of influences from sources of irritation, having a degree of permanence, must be less promising; and those resulting from irritation of the origins of the nerves in the brain or spinal cord, must be most unpromising of all.

The treatment in these cases must, obviously have reference to the removal of the irritating cause, whether acting as an incitant of hysterical perversities, by reflex influences, transferring the result of irritation from the different nerves in another situation, to the efferent nerves going to the muscles affected, or existing at the origins, or in the course of the nerves supplying the muscles which are producing the disproportionate force.

The primary treatment must evidently be medical, but the permanent results may require systematized movements and mechanical appliances, partly to assist the elongated muscles of the convex side in regaining their volume and shortening their length, and partly to tire out and lengthen the contracted muscles.

The division of the rigid muscles may be necessary. Time may be gained by this means for righting up the spine, by lateral pressure, and securing the commencement of change of nutrition of ligaments, shortening those on the convex side and lengthening those on the concave side, so that when the cicatrization of the divided muscles restores their functions, the antagonist muscles may have been developed for effectual opposition.

The results of this operation upon the spinal muscles are better explained, in many instances, by the supposition that a profound impression is produced upon the nerves analagous to that of the moxa, the actual cautery, and that of the galvanic puncture, all of which may, in some instances, be found to be valuable remedies. This theory, however, would always lead to the employment of the latter remedies, rather than the division of the muscles. In the division of the sterno-cleido-mastoid muscle, for wryneck, however, more may be accomplished, for, by a free suppuration, a proper connecting cicatrix may be obviated, or if formed, it may be too thin to draw the two divided ends of the muscles together, especially if the antagonizing muscles are aided by appropriate apparatus.

All the other muscles concerned in producing lateral deviation of the spine must completely re-unite after division, whether the incision is open or subcutaneous; and with all these other muscles the division must prove a failure if the disproportionate contraction of the muscular fibres continues. The break in the succession of the irritation arising in the spasmodically contracted muscles, and reflected upon themselves, is an element of the rationale of success in some cases of myotomy, practiced as a remedy for muscular contraction.

*Treatment of Confirmed Lateral Curvature.*

The words of LIONEL J. BEALE, in his work *On Deformities*, written more than thirty years ago, are peculiarly appropriate: "The great difficulty of treating this class of spinal distortions (lateral curvature) consists in the necessity of removing the weight of the body from the vertebral column, and at the same time using such exercises as will remove the evil by strengthening the muscles. * * * * Every day's experience proves to me the utter impracticability of removing a decided lateral curvature of the spine without mechanical extension. It would be as easy to reduce a dislocation of the femur without the assistance of art, as to correct a long continued deviation of the vertebral column without extension. In the earliest stages of the malady, exercise alone will remedy the evil, but we are seldom consulted until the curvature is confirmed and permanent; that is to say, it cannot be removed by any exertion of the patient, nor does it, as in the earlier stage, disappear while the body is in exercise and the muscles in action. If a child with incipient lateral curvature be watched during the time when she is engaged in any active and absorbing amusement, there will be no appearance of distortion, and at this period judicious management will prevent the establishment of a permanent curvature. But when the deformity has existed for a year or more, when it has become fixed in any degree, the ribs will have accommodated themselves to the deviation, and they will contribute to the maintenance of it; in these cases no variety of exercise, no kind of gymnastics, will do no more than prevent further mischief. To remove that which has become established, extension alone will be of advantage. The judicious combination of extension, exercise and repose will in time remove lateral distortions of the worst kinds, but without unremitting perseverance they will be of no avail, for it is not in a few weeks that good can be effected in such cases, months and even years are required. * * * * The time required for the cure of spinal deformities of course varies. During the period of growth, perseverance will almost invariably insure success, and if the distortion is recent the shape will be restored in a few months; but the exercises must be continued *afterwards*, to prevent a relapse. Even when the growth has ceased, if no absorption has taken place in the bones, if they have not yet been rendered cuneiform by pressure, we may sometimes succeed in removing the deformity entirely; but it is obvious, that when the osseous system is much implicated, a complete cure cannot be effected, and some degree of deformity must remain for life."

Dr. BEAL'S theory has been carried out by improvements in appliances by Dr. H. G. DAVIS, whose language in a paper published in the *Boston Medical and Surgical Journal*, vol. 46, No. 5, p 96, for March, 1852, is here quoted:—

"After several year's experience in treating curvatures by apparatus and by various modes of exercise, the conviction was forced upon me that in order to do it with any degree of assurance, it was necessary that the apparatus should be so contrived that it would not only remove the deformity, but it should at the same time leave all the muscles free to act, and the patient under the necessity of using them to balance and support the body as fully as without the aid of such appliances; also, that it should be so planned that it should effect as much during the sleeping as the waking hours. * * * * For the furtherance of the recovery the instrument should not be fixed or stationary in its adjustments, but should possess elasticity, so that if the form yields, the apparatus may follow up the change, and exert nearly as much force upon the curve as it did before it had yielded in any degree."

The introduction of elastic rubber was early seized upon by Dr. Davis, with which, in great part to meet this necessity for elasticity, to which he gave the name of "artificial muscle."

The *desirableness* of elasticity in the appliances was appreciated long ago. The late improvements have been made rather in the achievement of the desideratum than in the conception of the want. Yet, up to this time, nothing has been devised which will do away with the necessity for the horizontal position in the treatment of confirmed cases. It is indeed probable that when time enough can be devoted to a patient the hands of the operator and his assistants, with some very simple appliances, will do as well as expensive and complicated apparatus. In this direction are the "*Antiplastic*" *Movement of Werner*, (quoted from *Bauer's Lectures on Orthopedic Surgery*, *p.* 94.)

"The patient is placed upon a covered table in a dorsal position; the hands of the operator are employed to correct the deformity, and to bend the spine over in the opposite direction; and, in fine, the patient is directed to maintain the same for an hour or so at a time. Another competent person may take the place of the physician to facilitate proceedings, which should be repeated several times during each day."

*Mechanical Appliances.*

These are of two classes, those which produce direct extension, and those which act laterally upon the spinal column. The simplest of the first class is Darwin's Bed, in which the head of the bed is elevated about 12 inches, and the cap in resting the head is fastened to the headboard. The tendency of the body to slide downward makes the extension. The appliances for

(Fig. 14.)

(Fig. 16.)

(Fig. 15.)

lifting in the erect posture rest upon the pelvis, and receive the shoulders upon crutches; and where the head is to be supported a collar applies to the base of the skull and of the lower jaw. Dr. EDMUND ANDREWS, of Chicago, has published plans of apparatus upon this principle, moulding a sheet of copper over an accurate cast of the pelvis, and extending a rod up behind the spine, and by means of attachments to this rod, lifting upon the head or shoulders, or both.

See *Chicago Medical Examiner*, for September, 1863.

HAZZARD is said to have been the first to attempt to support the spine by apparatus fitting the trunk, and producing lateral pressure and counter-pressure, while the patient is in the erect posture and walking about. This, and the modifications of it, are now known as Tavernier's Belt. Figure 14, from BAUER, illustrates this, and also the crutch arrangement, for the direct lift from the pelvis. The principle is to apply pressure and counter-pressure upon the projecting or convex portion of the skin. The movable arrangement at the base of the upright implies that the pelvic piece is fixed in its position.

Figure 15, from TAMPLIN, is another modification of Tavernier's Belt, with the crutch added on the side of the depressed shoulder. In this plan, also, the pelvic band must be fixed in its position, to sustain the crutch.

Figure 16, shows the modification of this apparatus, by Dr. HENRY G. DAVIS, of New York. The belts are made of rubber elastic webbing, for ease and efficiency. In this plan, the pelvic band is smaller, and is not designed to give any support, but only to keep the upright shaft in proper position.

The appliances of both kinds, hitherto published, require to be made by skilful workmen, generally found only in the large cities. I have made an endeavor to devise a plan, which can be executed by any ordinary mechanic, and by any physician, who will give his time to the work. The framework of this apparatus is sufficiently illustrated by Figure 17.

*Explanation.*—A metallic frame, for lateral curvature, which may be extemporized without the aid of a skilled instrument

(Fig. 17.)

maker. *a a* Two uprights of hoop iron, or, (better,) of thin steel. *b b* Base of copper, tin, or sheetiron, reaching two-thirds around the pelvis, to which the uprights are attached. *c c* Two pieces of hoop iron, or of thin steel, for the side on which the shoulder is elevavated. *d* A thinner and narrower piece of steel, to pass under the armpit of the depressed shoulder. This is movable at the rivet attaching it to the uprights, and upon the distal end is attached an elastic strap, which passes up in front and over upon the prominent shoulder, and down to a buckle attached to the base *b b*, in order to make the lift under the depressed shoulder, and the weight upon the opposite elevated shoulder balance each other. *e* A strip of hoop iron (or other soft iron) to apply to the neck and base of the skull. All parts are properly padded and armed with straps and buckles.

To retain the head, after division of the sterno-cleido-mastoid muscle for wryneck, and after plastic operations upon the neck, a frame, upon the same principle, can be easily made, prolonging the uprights *a a* to pass over the top of the head, and employing two neck pieces, *d.* The uprights are made short at the lower end, so as to bring the base *b b* around the lower part of the chest. The pieces *c c* and *d* are left off for this purpose.

In Figure 18, the apparatus is seen applied. It will be observed that it differs from the preceding plans, in making the prominent shoulder the centre of force, and, in the erect posture, the support, also, of the weight of the apparatus.

(Fig. 18.)

Figure 18. Apparatus for lateral curvature applied. *aa*, *bb*, *c*, *d*, *e*, The same as in Figure 17, properly bent to the form of the body and padded. *ff* An elastic strap attached to the front or distal end of the bar *d*, passing up in front, and over the opposite shoulder, overriding the loop *g*, and passing down behind to the base *bb*, where it is attached by a buckle. *gg* A loop encircling the prominent shoulder, and attached by an elastic strap to a buckle fastened to the base *bb*. *h* A small cord passing around one of the branches of the loop *g*, and attached to the upright, for the purpose of keeping the loop from slipping off the shoulder. By means of the strap *ff*, and the loop *g*, the apparatus is made self-sustaining, and it is not necessary that the base *bb* should have any nice adaptation to the pelvis.

The apparatus, as shown in the figure, is adapted to a curvature in which the left shoulder is prominent. The bar made into a hoop *cc*, and the right extremity of the pelvic base *bb*, also made into a hoop, passing around the right ilium, are intended to be nearly unyielding. The bar *d* moves with the ascent and descent of the right shoulder, and all the straps and fastenings are elastic. This is to give the muscles more freedom of motion, both for comfort and to permit that to and fro movement, which gradually secures yielding of both muscles and ligaments, without danger of inflammation or ulceration anywhere. A vastly greater amount of pressure can be borne with this yielding elasticity, than without it.

The details of the apparatus admit of a great variety of modifications, to suit different cases. If it is desirable to support the head, to give the cervical vertebræ some extension, the weight of the head may be made to apply to the projecting shoulder, through the strap *f*, and the loop *g*, which pass over it. It is believed that this plan admits of a greater variety of application than any other which has been recommended, at the same time that it can be made from the most common materials, requiring very little skill.

The employment of the best steel and the best workmanship, to make the whole apparatus light and elastic, is desirable, but not necessary to success.

Figure 19, illustrates a case belonging to the second division of the classification here adopted—lateral curvature occurring in a lad, 17 years of age, of previous good health. The deformity has been chiefly produced during the last six months, and is observed to be progressing rapidly.

(Fig. 19. Wm. Richard Gore.)

He fell from a horse, three years ago, striking upon his head, and was nearly helpless for a few days after this. This is the only known cause. There is very little deviation in the lower dorsal, and in the lumbar vertebræ. Stature 5 feet.

After ten days use of apparatus delineated in Figures 17 and 18, the patient's height had increased half an inch.

In addition to mechanical treatments, the patient takes, three times a day, a teaspoonful of syrup of iodide of iron and simple syrup.

*Aug. 3d*, 1864, (about two months from the commencement of treatment,) increase of stature since last measurement, $\frac{5}{8}$ inch—whole increase, $1\frac{1}{8}$ inch. *Nov.* 5. Additional increase of stature, $\frac{3}{8}$ inch—total increase, $1\frac{1}{2}$ inch. At this point, there seemed to be a cessation of supposed softening of bone, and a fixedness of position accrued, so that no further progress was made. The general health improved. Perhaps greater amendment of form might have been secured, by requiring the patient to keep the horizontal posture, but it seemed necessary to permit him to move about, in aid of his general health.

To the medicine, is greatly due the credit of the arrest of the softening of bone, but the mechanical support retrograded the deformity before the medicine had time to act. At a later period, the restoration of bony firmness rendered further amendment of form impossible.

C. *Antero-posterior, Vertical, or Angular Curvature.—Potts' Disease.—Kyphosis.*

This curvature depends upon disease of the bodies of the vertebræ, resulting in destruction of the substance of the bone, while the articulations maintain their integrity. As the weight of the super-imposed parts forces the adjoining surfaces of the vertebral bodies together, the spinous processes must separate and project backward, with a more or less sharp curvature.

The pathology does not differ from that of inflammations and degenerations existing in other spongy bones, as chronic sequels of inflammations, following injuries, or as degenerations of spontaneous origin; the localizations of constitutional tendencies, directed to this seat by any cause diminishing the proportionate vigor of nutrition of the spinal bones. The freedom from acute pain, probably, depends upon the low grade of inflammation giving time for a slight expansion of tissue, to accommodate the increased amount of blood, and saving those nervous extremities from distention, which, when irritated, are susceptible to painful impressions. A dull, ill-defined, aching sensation, instead of an acute pain, usually attends the early history of the cases.

A careful step to avoid jars of the body, and a slightly stooping posture, with a disposition to brace up the trunk by placing the hands upon the thighs, and a careful avoidance of all those attitudes which bring increased weight upon the bodies of the vertebræ, are characteristics which mark the progress of the disease. In picking up objects on the floor, the patient carries his hand to the floor by flexing the hip and knee-joints, holding the spine stiff and nearly erect; a movement so contrary to that of persons with healthy spine as to attract attention. Rheumatism may occasion constraints of movements very similar, and hence a particular caution is necessary in distinguishing osteitis of the vertebræ, until the persistence of the symptoms renders the diagnosis plain.

From the movements of the patient, we may see that there is an effort to save the vertebræ from pressure, though the sensations are so diffuse that the patient is not aware of the seat of the uneasiness; those startings and spasmodic muscular contractions which attend inflammation of bones adjoining more moveable joints, are usually absent, owing to the absence of the friction of rough surfaces, such as apply to each other in destructive inflammations of the knee and hip-joints. The patient is able to save the bodies of the vertebræ and the intervertebral cartilages from pressure while in the erect posture, by active contraction of the erecting muscles of the spine attached to the spinous processes, but they soon tire out and yield to the tendency of the spine to curve, just as muscles elsewhere yield to an overcoming force applied by extending apparatus. This yielding becomes habitual and permanent with increased length of muscles. As this process goes on, the *ligamenta subflava* connecting the arches of the verterbræ become permanently elongated by change in nutrition, while the articulating process assume new relations with each other. An abrupt projection thus occurs in marked contrast with the curves of the lateral deviations. No tenderness on pressure usually attends this process of destruction, as the diseased parts are beyond the limit of the influence of pressure upon the spinous processes, upon the ribs, or upon the adjacent muscles. The amount of destruction of bone is truly astonishing; the bodies of several vertebræ in some instances disappearing. A striking case of this kind is given in *Cruventhier's Pathological Plates*, 4th livraison, plate 4, in which there was a loss of the bodies of five vertebræ, the 5th and 11th dorsal vertebræ coming in contact by their anterior surfaces, the spinal canal making an acute angle, without the occurrence of paralysis at any time. The illustration of this case is copied in figures 20 and 22.

(Fig. 20.)

"The individual to whom this specimen belonged was not paraplegic, although the angle was so acute, but the pressure of the spinal marrow was probably prevented by the meeting of the 11th with 5th dorsal vertebræ, which furnished a groove for the reception of the 11th. The bodies of the 6th, 7th, 8th, 9th, and 10th dorsal vertebræ have almost entirely disappeared, their confused remains forming a mass covered with oseous vegetations. The foramina were all preserved, but deformed and diminished in size. The spinous processes corresponding to the last vertebræ had undergone a remarkable deviation; instead of projecting more than usual, they were much inclined and even slightly curved so as entirely to fill up the interval which separated the 8th from the 9th, and the 9th from the 10th dorsal vertebræ and to complete posteriorly the medullary canal. The manner in which Nature had preserved the spinal canal in the midst of such devastation is remarkable. The vertebral column having been sawed lengthwise we see (Fig. 21) with what apparent art the canal had been protected."

(Fig. 21.)

The occurrence of pressure upon the spinal cord is obviated by the slowness of the changes and the ultimate incasing of the cord by new bony material coincident with the anchylosis of adjoining vertebræ, except in those cases in which the weakened bony substances are fractured by the weight of the parts sustained, or by some movement or force suddenly applied greater than the vertebræ can endure. By this sudden fracture, the spinal cord may become compressed by the fragments and paralysis ensue. By the absorption or disintegration of these, or by their change of position through extension acting through the spinal column, the pressure may disappear, or through failure of these the pressure and attending palsy may be permanent. The duration of the disease from its incipiency to the termination by anchylosis is measured by years. During the period of aggravation pus may form and accumulate in such quantity as to burrow through the adherent pleuræ into the lungs or along the fasciæ of the muscles into the lumbar region, or along the psoas muscle into the groin. Cases, however, may doubtless be cut short previous to the suppurating stage, while little or no deformity has ensued, as in similar inflammations in other cancillous bones. It may not then be apparent to the patient or friends, or even to the physician, what is the magnitude of the evil cut short.

There is a doubt thrown upon the efficacy of treatment in this and other diseases pursuing such a protracted course, because the disease does not at once begin obviously to ameliorate upon the institution of treatment. From analogy and experience, however, the following indications may be safely affirmed:

1. The disease, except when it is tubercular or a cancerous degeneration, has an acute stage in which the treatment must be like that in the acute stage of other diseases of general low grade. This element of treatment is important before the bony substance is so absorbed or disintegrated that the pressure of the weight of the parts above in the erect posture can have any important influence. Whether the morbid processes have their origin in an injury or in some obscure constitutional influence localizing itself in the spine by an accidental selection, the case should be treated on the same principles as strumuous inflammation in the eye. General heroic treatment cannot be borne, but active purgation once a week, cleaning out the colon and stimulating the liver and other alimentary glands, creating an appetite and giving a healthier hue to the surface, will constitute an important element in the treatment. Mercury is not an indispensable element in the purge, but it certainly adds very much to its efficacy. A grain of calomel for a child 5 or six years old, taken at night, and followed in the morning with an efficient senna draught, may be mentioned as an appropriate purge. The mercurial may, for convenience, be combined with a sixteenth of a grain of tartar emetic, two grains of leptaudsin, and two grains of sugar, for a patient of this age. Where the mercury is omitted, two grains of leptandsin may be given at night, followed by castor oil in the morning. Should there be considerable febrile excitement, the force of the circulation may be diminished by small and frequently repeated doses of tartar emetic, or in more active cases, veratrum viride. This element of treatment may be expected to be of most efficacy while the diagnosis is obscure, the disease being suspected by the gait and posture of the patient, before any deformity is manifest. It may be said in favor of the treatment that if the diagnosis is wrong the remedies are equally appropriate for the rheumatic stiffness which simulates the spinal disease.

2. Following this class of remedies, or in connection with them, the employment of iron and iodine, with or without other tonics, may be expected to have an important influence in overcoming the constitutional perversion upon which the perpetuation of the local disease so much depends. The syrup of iodide of iron, of the U. S. Pharmacopœia, in doses of fifteen drops three times a day, for a child six years old, may be instanced as a convenient and efficient form of prescription. It is important to insist, that when there is any fulness of bloodvessels felt in the pulse, or seen in the capilliaries of the surface, this class of remedies should be preceded or accompanied by a proper eliminative treatment. It is feared that the present prevalence of the humoral pathology in medical theories, too often leads to a neglect of this important principle of treatment to prepare the system for the absorption or tolerance of iron tonics.

3. Local remedies; cupping and leeching, emollient applications, the most convenient of which is a damp towel persistently worn over the spine, and moistened as often as it dries, slightly to reduce the temperature of the skin, and to soothe the nervous extremities. This may be supposed to act chiefly by reflex influence, being applied to nerves having origins near to the origins of those going to the diseased parts, or identical with them. Blisters, moxas, and canteries, however, if employed in this stage, should be applied at a distance, so as not to excite an increased sympathetic activity in the diseased parts. It is not impossible that the disrepute into which these last remedies have fallen is owing to the disregard of this therapeutic principle by the old surgeons, who applied these remedies too indiscriminately. Observing their efficacy in the later stages of the disease in which spasmodic and painful muscular action had been sympathetically produced by irritation of the nerves of the diseased tissues. A hasty generalization may have led to their too early employment and to their application too near the diseased tissues. It is hardly possible that their employment should have been an entire mistake. The extent to which Brown-Séquard has insisted upon this latter point will doubtless have a controlling influence upon future practice. A practical question will often arise, whether a purulent accumulation in the groin, or elsewhere, should be opened? The elements of the answer to this question may be thus stated:

Pus enclosed in the tissues and kept from exposure to the air, whether in large quantities or small, is capable of maintaining its original composition for a long period, and of being finally absorbed. Undecomposed pus is unirritating to the tissues, does not interfere with healing processes in contact with it, and travels chiefly by the pressure of its own weight, or of its bulk when in too confined a space, as in phlegmonous diseases, in which pus rapidly accumulates. The presence of undecomposed pus, therefore, will neither aggravate the disease nor prevent the healing of the abraded surfaces of bone and cartilage. On the other hand, the complete evacuation of the pus is impracticable in consequence of the tortuousness of the passages or the formation of pockets from which the pus can only be slowly drained by position. The admission of air to a portion of the pus within the tissues, sets to work a process of decomposition which gradually communicates to the whole. This inflames the interior of the pus-holding sack, and the tissues originally diseased may take on an aggravated form of inflammation from this new source of irritation. The attempt to evacuate the pus through a tube ending under fluid can never evacuate the less liquid portions of the pus which are liable to block up the tube, and if successful in avoiding the introduction of air into the cavity, the wound must heal by the first intention, to avoid the unwelcome result of decomposition of pus and consequent irritation. If an opening has spontaneously formed into the air-passages of the lungs, or in any inconvenient location, the case can be no worse by the making of a free opening in the most dependent position, the latter to drain the parts, and when there is an opening into the lungs, to save the air-passage from the burden of this discharge. The introduction of local medicines by injecting them into the purulent cavity, other than disinfectants, promises too little to encourage the practice.

After the formation of pus has ceased in the progress of restoration, and after the parts originally diseased are well covered by granulations, reducing the case to the condition of an ordinary chronic abscess, a state of things indicated by the greatly improved state of the patient, and the increased strength of his back, there can be no more objection to the evacuation of the pus than in an ordinary chronic abscess.

4. Quiet of the general system by confinement, chiefly in the horizontal posture, is of greater importance than parents can easily appreciate, both for obviating the general arterial excitement upon which the activity of the local disease in part depends, and for relieving the diseased parts from any functional action in sustaining the weight of the body. Whatever objections may be urged against the observance of the horizontal posture in the later stages of the disease, in the period of wasting from suppuration, and in that of repair and progressing anchylosis, none can be urged on therapeutical grounds against the observance of rest and posture during the early period of subacute excitement, while tonics cannot be borne unless preceded by eliminants. Indeed, it is probable that if this indication could be properly appreciated by parents and physicians in the forming period of the disease, vast numbers of cases would recover without proceeding so far as to afford a clear diagnosis, and of course without any deformity.

5. Artificial support of the diseased vertebræ, taking off the pressure upon the bodies, and obviating the strain upon the muscles and ligaments attached to the spinous processes and arches of the vertebræ, is seen at a glance to be theoretically correct. So far as the disintegration of bone and intervertebral cartilage may depend upon pressure, the relief of this pressure becomes an important element of treatment. The older orthopedists attempted to meet this indication by direct extension upon the spinal column, by support applied to the base of the skull, for disease of the cervical and upper dorsal vertebræ; and for the middle and lower dorsal and the lumbar vertebræ, by crutches resting under the armpits, and attached below to a metalic support resting upon the crests of the iliac bones, properly padded and retained by a strap passing across the abdomen. It is believed that Dr. HENRY G. DAVIS, of N.Y., is the first to have employed the vertebral column itself as a lever to straighten it or to keep it straight in Potts' disease; relying upon the articular processes as fulcroms upon which the force of the extension comes. This expedient of treatment is founded upon the law of the disease to affect the bodies of the vertebræ, and to leave the arches, articulating surfaces, and processes untouched. The principle of treatment is that of a splint applied to the back of the spine to keep it from bending, and to force the weight of the parts above upon the articulating processes.

In the No. of the *Boston Medical and Surgical Journal* for August 4th, 1852, Dr. DAVIS, at the close of an article on lateral curvature, refers to an apparatus for Potts' disease, having then employed it for three years. In the *American Medical Monthly* for 1856, vol. 5, *p.* 212, &c., Dr. D., in speaking of the difficulties in adapting remedies for Potts' disease, remarks: "The common mode of constructing apparatus to sustain the weight of the body upon crutches is utterly useless. * * * The bodies (of the vertebræ) and the oblique processes afford the only perpendicular support. The distortion is produced by the removal of the bodies of the vertebræ by ulceration. As the line of perpendicular support falls between the bodies and the articulations of the oblique processes; the weight of the trunk above approximates the bodies of the two adjoining vertebræ, as the diseased one is removed by absorption; the oblique processes now sustaining the weight of the trunk act as fulcrums upon which the vertebræ are tilted or rotated; thus, the spinous processes above and below are separated from that of the diseased vertebræ, the articulations of the oblique processes being the centres of motion. It is this form of the vertebræ which enables us to make use of the whole vertrebral column as a lever to restore it. By apparatus we are enabled to throw the entire weight of the superincumbent body upon the oblique processes, and at the same time separate the bodies of the vertebræ adjoining the diseased one from it, the contact of which is constantly irritating and producing absorption. * * * The same principle of treatment, viz.: the separating of the diseased surfaces, and removing from them all irritation from pressure, is equally applicable to diseases of the hip-joint."

These quotations are made to this extent not only to vindicate American Surgery against foreign claims, but to secure the honor of originality to whom it rightfully belongs among Americans.

In the *Transactions of the New York State Medical Society* for 1853, is an article by Dr. C. F. TAYLOR, of N.Y., upon "The Mechanical Treatment of Angular Curvature," &c., which has been reprinted in a pamphlet of 48 pages by Bailliere Bro's, in which Dr. TAYLOR claims that he is the inventor of the only method which does not rely upon crutches to support the body in Potts' disease. From a letter from Dr. TAYLOR himself, we learn that his own apparatus was invented in 1859. This is several years after Dr. DAVIS had employed his apparatus. The latter has no hinge, and secures itself above by pressure upon the sternum and clavicle; while TAYLOR'S apparatus makes fast to the shoulders by bands which encircle them, one on each side.

The hinges in Dr. TAYLOR'S apparatus must certainly be useless complications of machinery, for the very object of the apparatus is to obviate movements of the vertebræ upon each other, and not to make special provision for motion. The elasticity of the material affords all the motion which should be permitted. In theory, it would be correct to abolish all motion of the affected portion of the spine.*

(Fig. 22.)

Figure 22. Apparatus of Dr. H. G. DAVIS, for vertical curvature, arranged for disease of the lower dorsal, or upper lumbar vertebræ.

Dr. LOUIS BAUER, of Brooklyn, has constructed a modification of this apparatus, which he calls a cuirass. It is made of woven iron wire, by fashioning it upon a plaster cast, and encircling its borders with an iron rod or wire, of sufficient firmness to resist any force likely to be applied to it. The fastening above, is by bands around the shoulders, and below, by a band across the hypogastrium. On either side are leather handles, for the purpose of carrying a smaller patient invested with this covering, like transporting a turtle on his back.

The apparatus is made like the "wire breeches" of the same inventor, and, when made a good fit, it must answer the purpose admirably.

It is a desideratum, to have a plan which can be executed anywhere, away from mechanics especially skilled in the making of such appliances. To this end, let a strip of hoop iron be cut, of sufficient length to extend along one side of the spine, and another like it, to extend parallel with it along the other side,

* The following advertisement has appeared in the advertising department of several medical journals:—

"The Mechanical Treatment of Angular Curvature, or Potts' Disease of the Spine. This new method of treatment, first brought before the profession through the *Transactions of the Medical Society of the State of New York*, and attended with such marked success, is here offered in pamphlet form, convenient for transmission through the post. Price 35 cents. BAILLIERE BRO'S, 520 Broadway, New York."

to be separated, so that the spinous processes shall be free from pressure. Let the lower ends of these strips be riveted to a wider strip, which may be made of tin, to encircle one-half the body, passing across the upper part of the sacrum and iliac bones. A broad strap, to fasten with a buckle, is to invest this piece of tin and encircle the body. The lower ends of the vertical strips are thus held fast to the pelvis. The upper ends are to have a short cross-piece riveted to them, with an attachment at each end, for a padded strap going round the shoulder, with a short elastic at its fastening to the splint, to relieve the shoulder of the discomfort of an unyielding constraint, as employed by Drs. TAYLOR and BAUER, or the strips may be originally cut longer, so as to be bent in the form of a hook, passing over the clavicles in front. To give breadth of application to the ribs, let a piece of tin or sheetiron, of sufficient size, one for each side, receive two transverse slits, through which the vertical strip is passed, before it is riveted at both ends. The proper form of this metallic splint may be secured by bending it across the knee, and ample padding should shield thinly covered projecting bones from pressure.

(Fig. 23.)

Plan for an iron framework for apparatus for vertical or angular curvature (Potts' Disease).

*a a* Two parallel uprights. *b b* Base to apply to the pasterior half of the pelvis. *c c* Plates of sheet-iron, or thick tin, to be padded, to apply on either side of the affected portion of the spine. They can be made to slide upon the uprights till they come to the proper place. *d d* Upper cross-piece, to each end of which is attached a loop, well padded, to encircle the shoulder. As neither elasticity nor any other motion is desirable, the uprights should be made of soft iron, in order to be easily adapted to the shape of the trunk, and of sufficient bulk not to yield to the tendency of the trunk to bend forward.

If the disease is in the upper dorsal vertebræ, it may be better to make the uprights *aa* long at the top, to bend over in front of the clavicles, in the form of hooks.

If the disease is in the cervical vertebræ, the plan will do better, which is recommended for lateral curvature, crossing the uprights opposite the neck, and supporting the head by a cross-piece which passes under the mastoid processes and lower jaw. (See Figure 17.)

If the disease is in the sacrum or lumbar vertebræ, it is doubtful whether any expedient but the horizontal posture will obviate deformity.

It is believed that, for efficiency, a homemade apparatus fashioned in this manner, may be equal to a more costly one. To bring the means of treatment within the reach of all, is the desideratum.

For disease of the lower lumbar vertebræ this apparatus is inapplicable, and one that directly lifts, like that of Dr. ANDREWS, of Chicago, can do but little good, on account of the impossibility of avoiding the disposition of the spine to bend forward. The difficulty is to extend the lower end of the lever sufficiently below the diseased bones, to prevent the inclination of the body to bend forward. Anchylosis of the hip-joints would afford the desired condition. A mere lift acts upon the bodies of the lumbar vertebræ at too great mechanical disadvantage. Nothing remains but the horizontal posture.

For disease of the cervical vertebræ, the upper end of the apparatus must take hold of the head. The most feasible plan for this purpose, is to employ strips of iron, as elsewhere explained, for lateral curvature, (Fig. 17,) crossing them between the shoulders, like shears, so that rests may be fastened to their upper extremities, to support the head and face. The constraint of movement may be very undesirable, but, for a patient that cannot otherwise hold his head up, it may be a very acceptable relief, as well as an efficient therapeutic agent. In this case, it is practicable to rest the weight of the head partly or entirely upon the shoulders of the patient, rendering it unnecessary that the apparatus should extend farther down than is necessary to give firmness of support to the splint employed as a lever.

6. Finally, the question arises as to the time, and the degree of exercise, and of food, whether the good effect of exercise on the general health will balance the local irritation from the unavoidable jar and friction of the diseased parts. The question is nearly answered by what has already been said. In the acute stage, and while the abortion of the disease is possible, quiet is desirable for its effect on the general circulation, as well as the avoidance of local irritation. Later exercise would be desirable for the general health, while it must be destructive by aggravating the local disease, and, through this irritation, increasing the general irritative fever. The extent to which the spine may be supported by apparatus, will then determine the propriety of exercise in the erect posture. Still later, when the destructive processes have ceased and the reparative processes have commenced, there is great need of the invigoration of exercise and open air, with the amusement of the senses by changing scenes, but, without adequate support to the spine, there is the greatest danger of breaking down the weakened column and exciting the inflammation afresh, or producing a palsy, by the pressure of the fragments upon the spinal cord. Here, no little value of a splint is the safety afforded, while the general health is invigorated by exercise in the erect posture and in the open air. Without this protection, the exercise, indoors or out, should be limited to such movements as can be executed in the horizontal posture, until, by cautious trials, it is found that the patient can endure shocks without uneasiness, and refrains from supporting the trunk by placing his hands upon his thighs.

The question of food is more simple, because the exercise of the gastric function produces no jar or friction of the parts diseased. During febrile excitement, food must be abstained from, simply because it cannot be retained and assimilated, as in a similar general state from any other local disease, but, as soon as the effects of eliminating medicine, rest, and the recuperative tendency render digestion possible, the introduction of nourishment is of the greatest importance, for a well-supplied nutrition is one of the most effective regulators of movements, perturbed by the unbalancing influences of inanition. Thus, a judicious succession or alternation of medication and diet may do what neither could accomplish alone. The power of the digestive organs must always be regarded, so that hardly any other rules can be given, than the regularity of meals and the avoidance of too long fasting. To this end, a cracker or a piece of bread at bedtime, may conserve the powers and render the breakfast more easily digested, than would be the case after twelve hours fasting.

The temporary use of alcoholic stimulants, to excite the gastric glands by direct stimulus, or through the excitement of the brain, may often be extremely useful, but the permanent employment of any of their forms, as elements of diet, could not be productive of any but pernicious effects.

D. *Strabismus.* In aggravated cases of strabismus, the division of the contracted muscles is usually satisfactory in its result, because, if it fails of complete restoration, it is at least an improvement on the previous condition. In some cases, more frequently in the less aggravated degrees of deformity, notwithstanding the greatest care, the deviation is overcorrected, and the pupil turns too far in the opposite direction. In other parts this result is never to be feared, because there are ready means of retaining the parts in any desired position, but in the eye there is no such means short of a suture in the conjunctiva. Great care in avoiding unnecessary division of areolar tissue, renders this overcorrection rare, but then, the frequency of failure of sufficient correction, and a necessity for a repetition of the operation have greatly reduced the favor in which this operation was held soon after its introduction by Stromeyer and Dieffenbach in 1838 and 39.

The success recently attending attempts to change the contracting distances of the internal and external recti muscles render it probable that the expedient of dividing the muscles will be reserved for those cases which resist the treatment by *exercise.*

*Exercise of the muscles of each eye separately, in order to give*

*the muscles varied capabilities, elongating shortened muscles, and contracting lengthened ones.*

This is most conveniently done by placing before the eyes a pastboard blind with large orifices opposite each eye. Across each of these orifices a slide, having a smaller orifice in it, is so placed that it may be readily moved from or towards the centre. Where only one eye is ordinarily employed in vision, the orifice over the best eye is entirely closed and the slide over the other is so moved that the poorest eye can only see by an extreme abduction. The exercise in reading and looking at various objects is continued until the discomfort becomes intolerable, and repeated several times a day until the contracted muscles acquire the power of elongation, and the elongated muscles the capability of contracting to a shorter length. It is difficult, if not impracticable, by this expedient to exercise the two eyes at the same time. It may be a useful preparation for the correct association of the two eyes, in looking at objects unaided by refracting medea, or by the aid of prisms which tend to produce double vision, tempting the patient to invert or to evert the pupils to avoid the appearance of two objects. The bases of the prisms placed toward the nose will tempt the patient to invert the pupils, and the bases toward the cheeks will make a temptation to evert the pupils, separating them wider apart.

*Stereoscope.*

In the *Philadelphia Medical News and Library*, for November, 1864, copied from the *London Medical Times and Gazette*, September 24, 1864, is a very brief reference to some experiments of Mons. Javal, of Paris, related in a Congress of Ophthalmologists at Heidelberg, in September, 1864, *in the use of the Stereoscope in correcting convergent squint.* The method consists in placing upon a card, cut of the ordinary size of those upon which stereoscopic pictures are placed, two slides (like a strip of paper around a package of envelopes) upon each of which a wafer is attached, both being at the same height upon the slides. This card, thus supplied with its wafers, is then placed in the position the picture is intended to occupy. For convergent strabismus the slides should be separated until two slides and two wafers are seen. Then by steadily looking through the prisms, the pupils diverge and the two images fuse into one. By the prolonged and frequently repeated exercise of the muscles of the eyes in this manner the parallelism is expected gradually to be regained.

### *Plane Prisms.*

In *Braithwaite's Retrospect, No.* 50, *January*, 1865, *Am. Ed.*, *p.* 158, is an article by ERNEST HART, Ophthalmic Surgeon to St. Mary's Hospital, London, copied from the *Lancet*, July 30, 1864, in which he gives an account of *the use of plane prisms* in correcting the deviation of the eyes in strabismus. He relates cases of succcess, and explains the result as owing to the effort to change the direction of the affected eye to avoid the double images which the prisms tend to produce. The principle is that of the stereoscope which causes two images to appear from a single central object, and a single image to appear from two objects placed at the proper distance from each other, and seen with both eyes at the same time. It is the effort of the muscles of the eyes so to direct the pupils that these double images may be avoided, which accustoms the shortened muscle to act in longer distances, and the elongated muscle to act in shorter distances, which by degrees secures a restoration of the proper parallelism of the eyes. The bases of the prisms are placed opposite the direction of the squint. So, for convergent squint or cross-eye the prism would be arranged as they are in the stereoscope, and in divergent squint in the opposite direction. The angles of the prism are made to vary according to the effects desired, using those of 4, 6, 8, 10, and 12 degrees, employing for constant use those of the degrees which only double the images at considerable distances.

Cases which are extreme are to be treated by division of the shortened muscles, and those which are sympathetic of still existing irritation or inflammation are to be reserved for treatment after the central or reflex irritation has subsided.

The treatment of strabismus without operation is in its experimental period, and all that is here attempted is to state the progress of experiment.

## Figures and Tables

**Fig. 3. f3:**
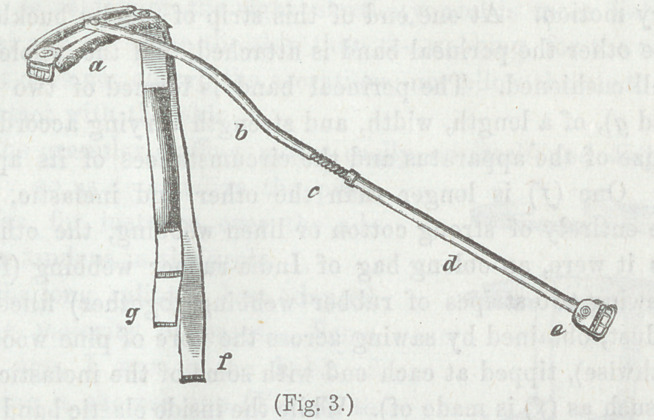


**Fig. 4. f4:**
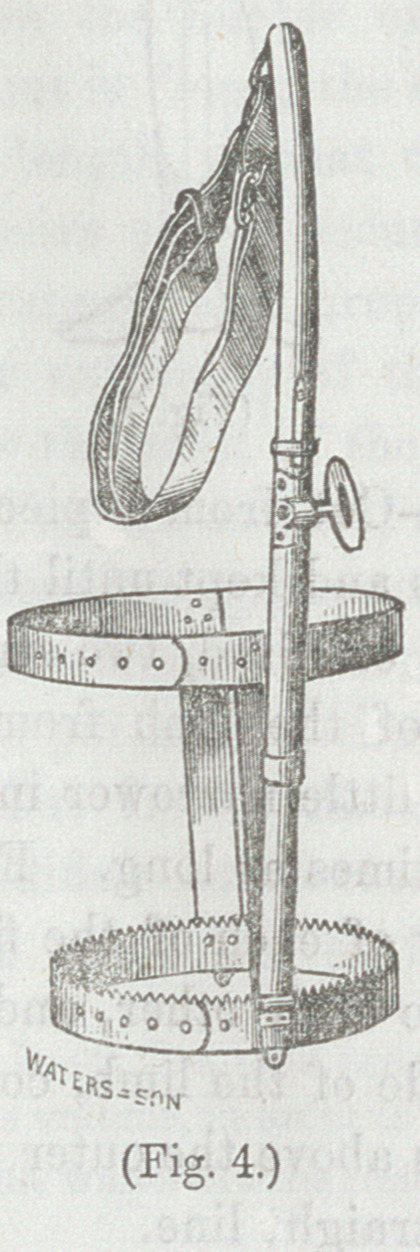


**Fig. 5. f5:**
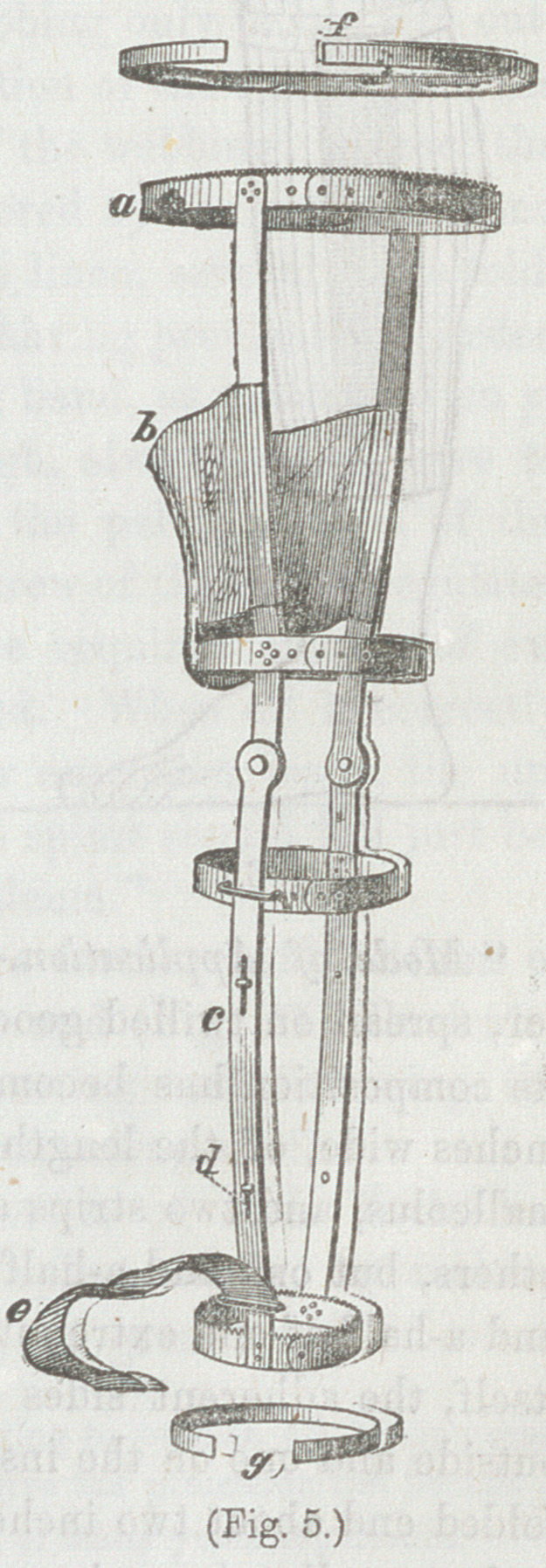


**Fig. 6. f6:**
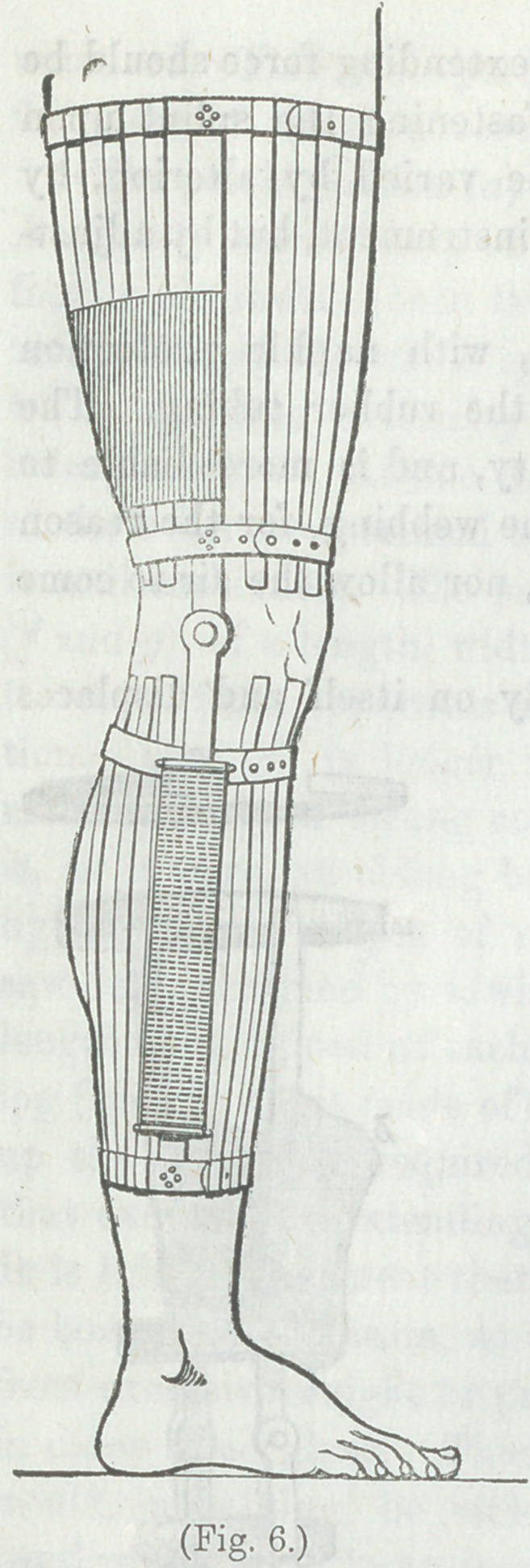


**Fig. 7, f7:**
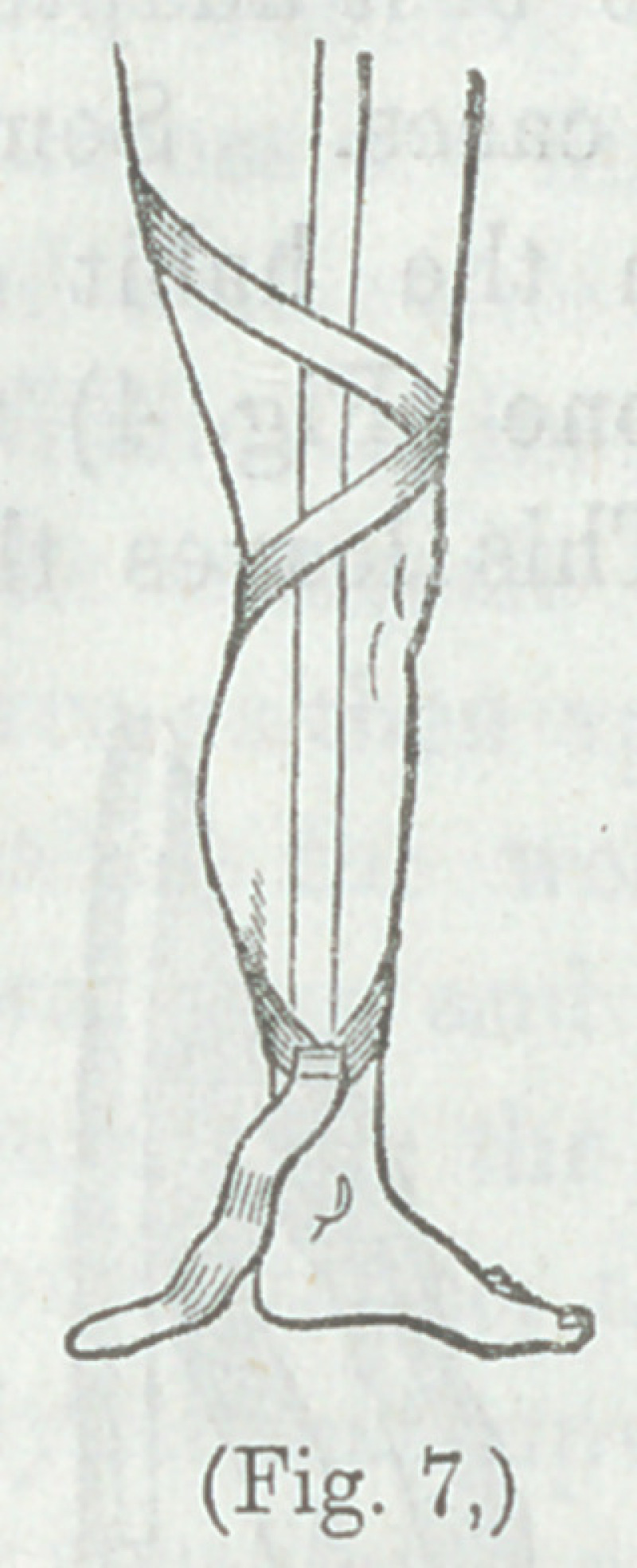


**Fig, 8. f8:**
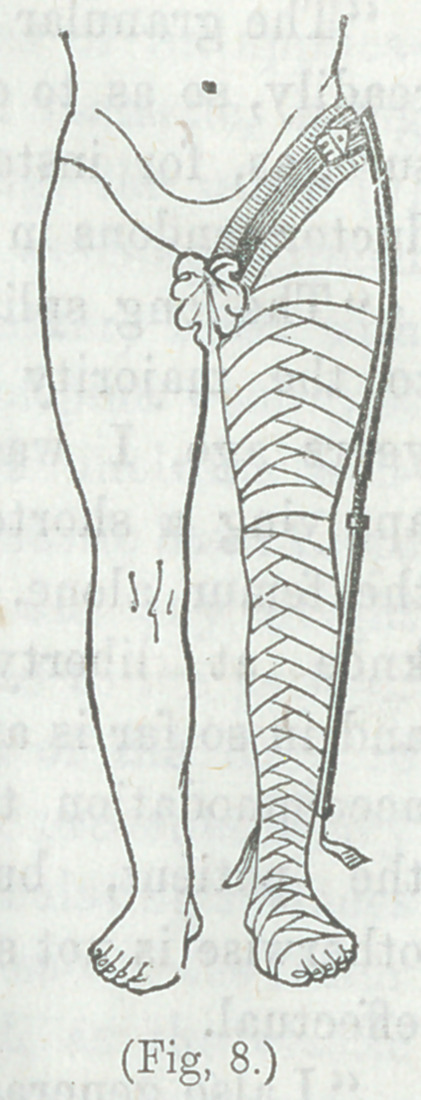


**Fig. 9. f9:**
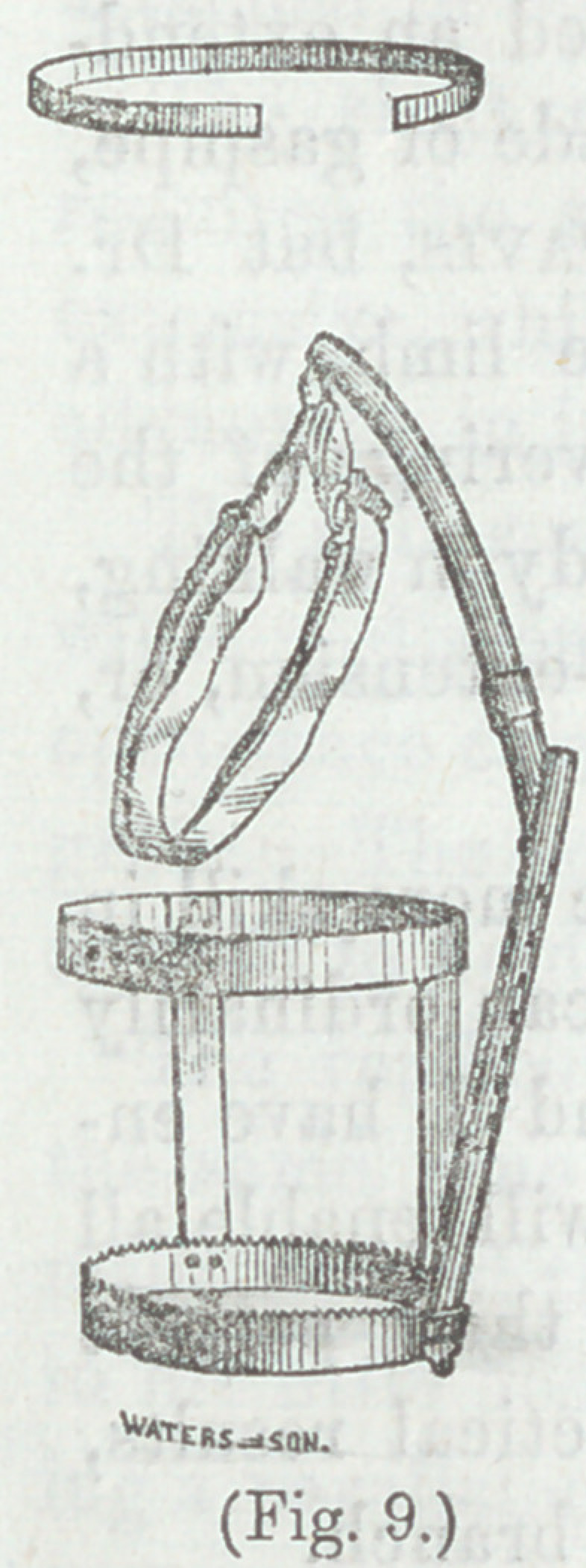


**Fig. 10. f10:**
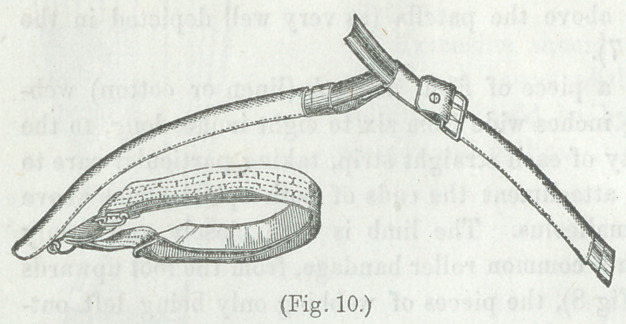


**Fig. 11. f11:**
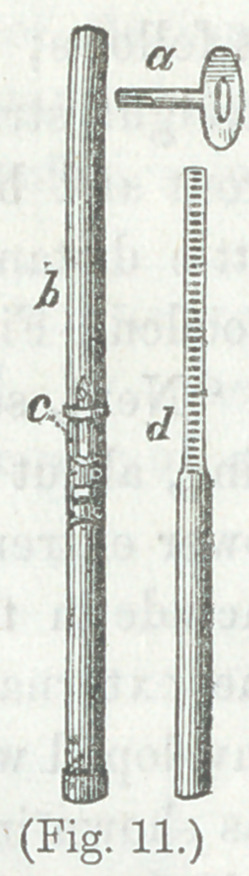


**Fig. 12. f12:**
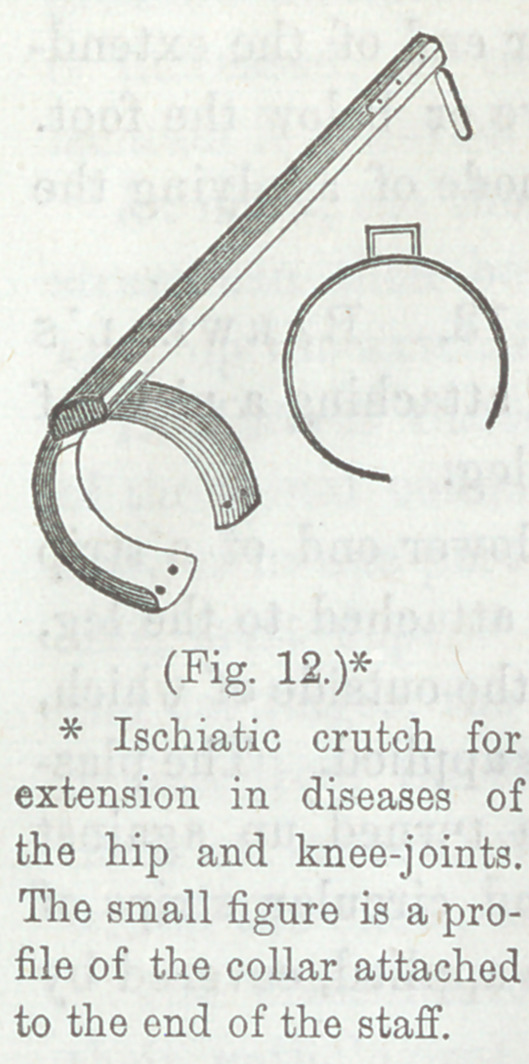


**Fig. 13. f13:**
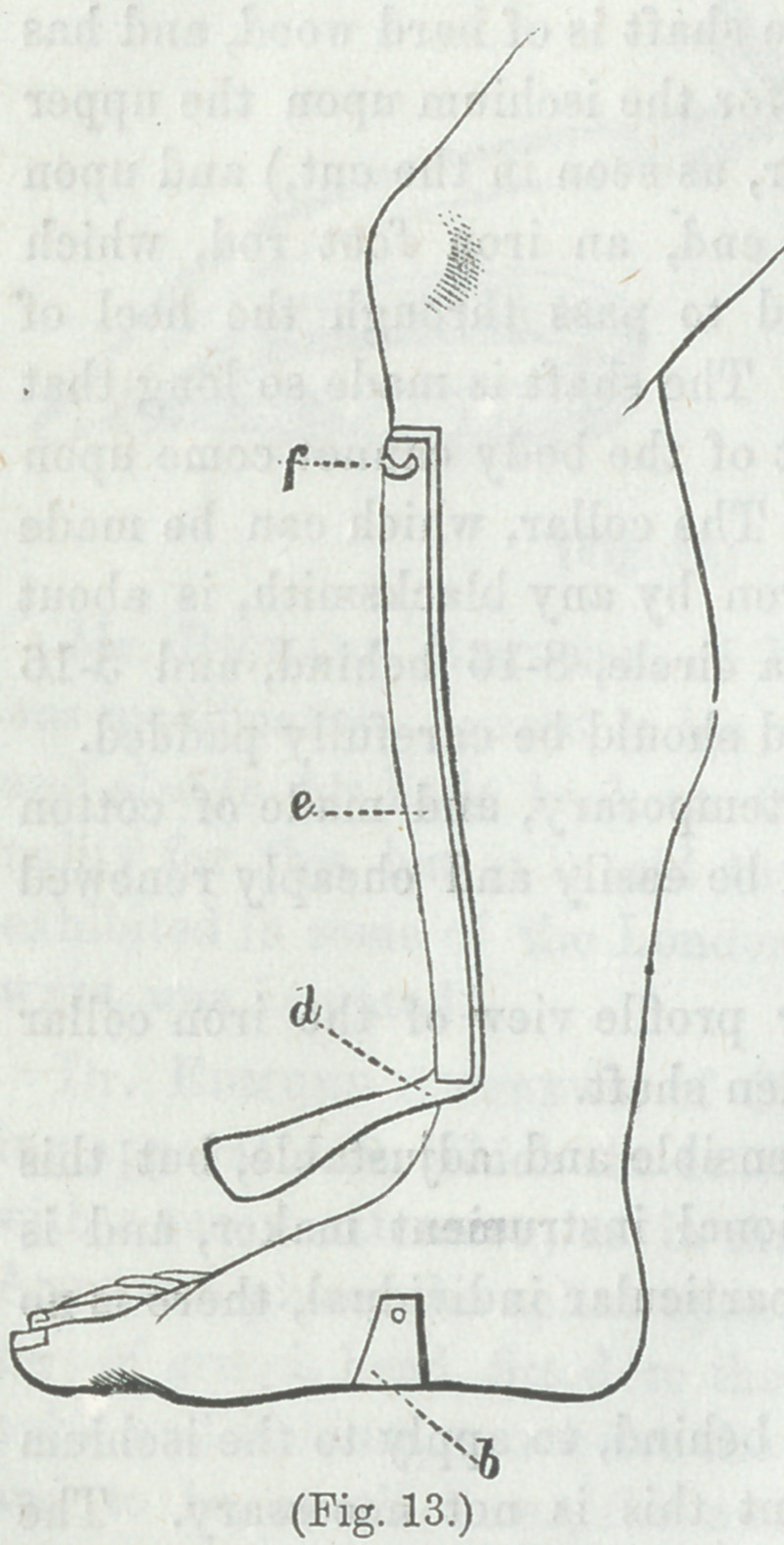


**Fig. 14. f14:**
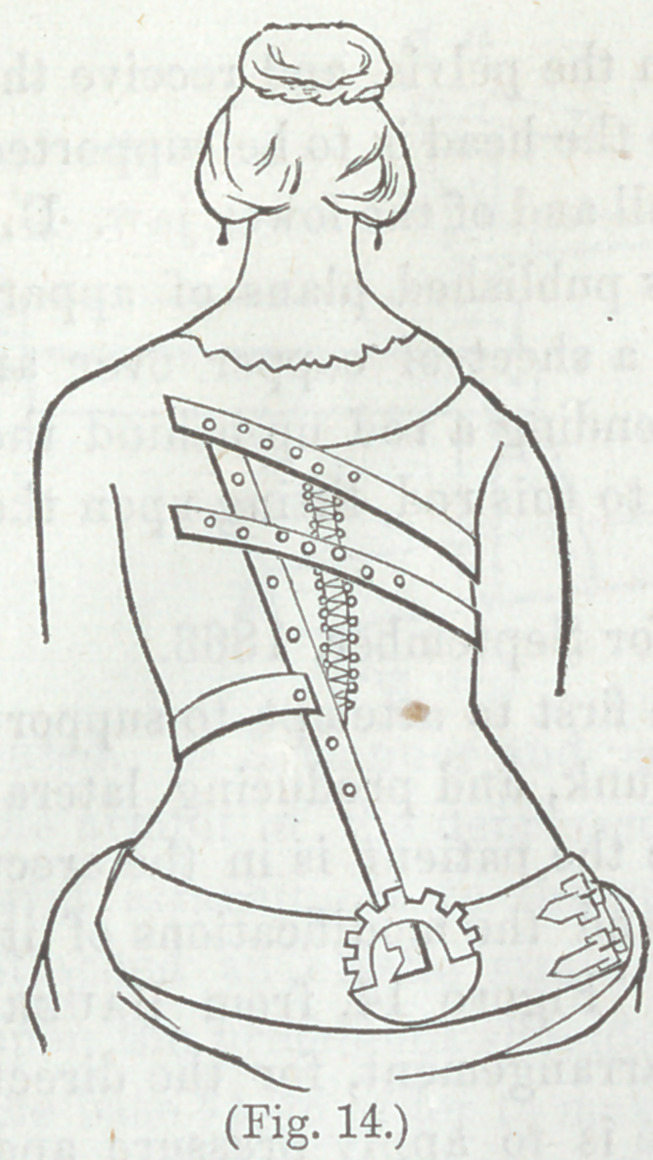


**Fig. 15. f15:**
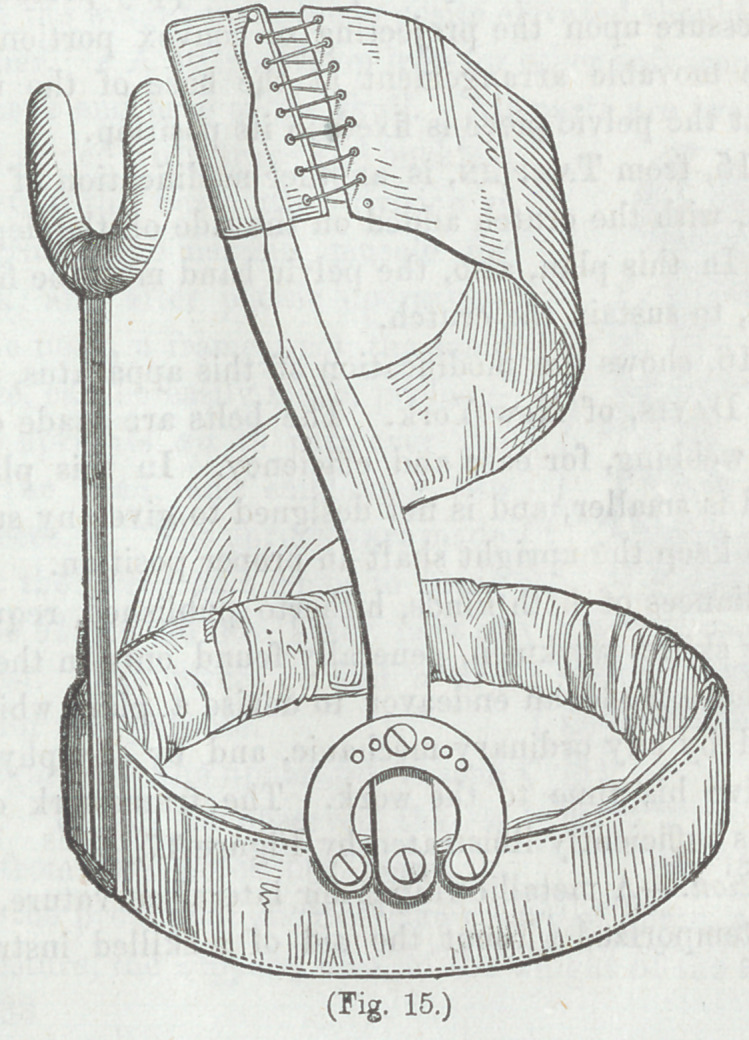


**Fig. 16. f16:**
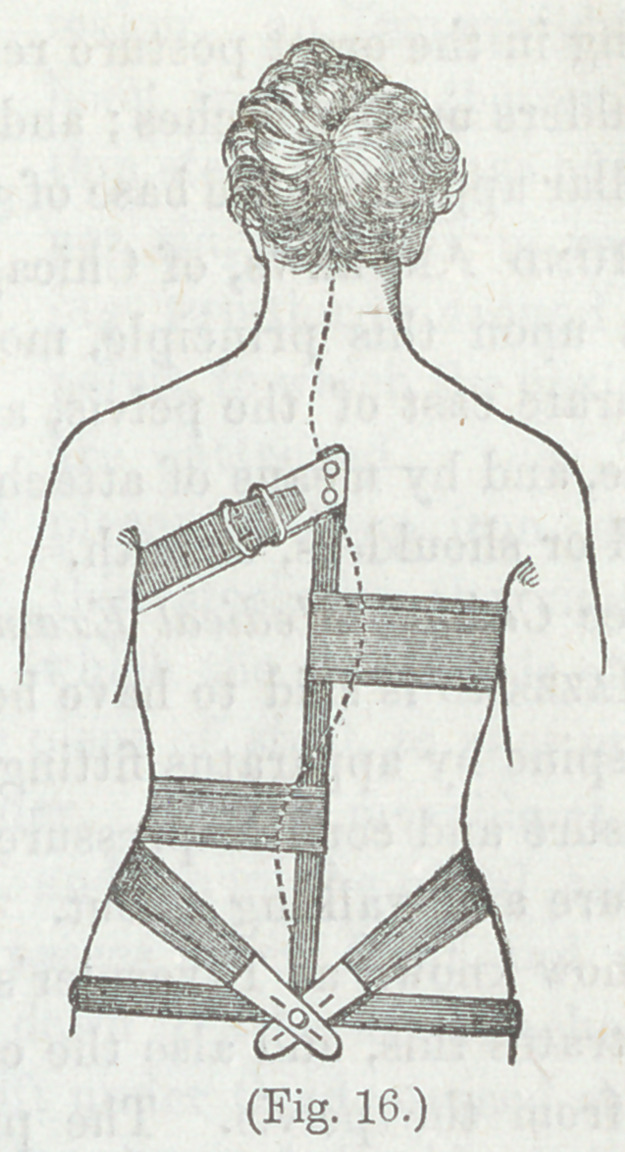


**Fig. 17. f17:**
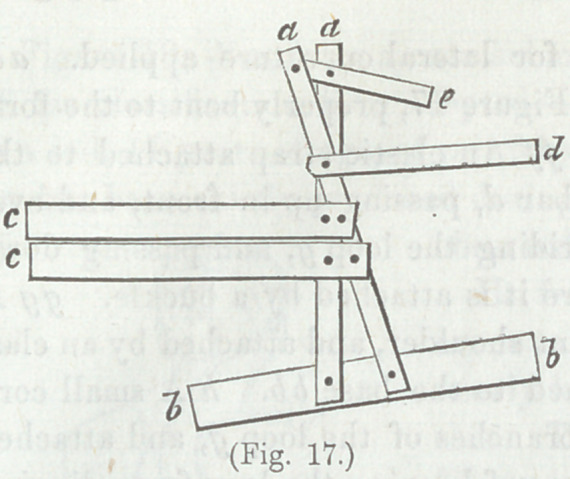


**Fig. 18. f18:**
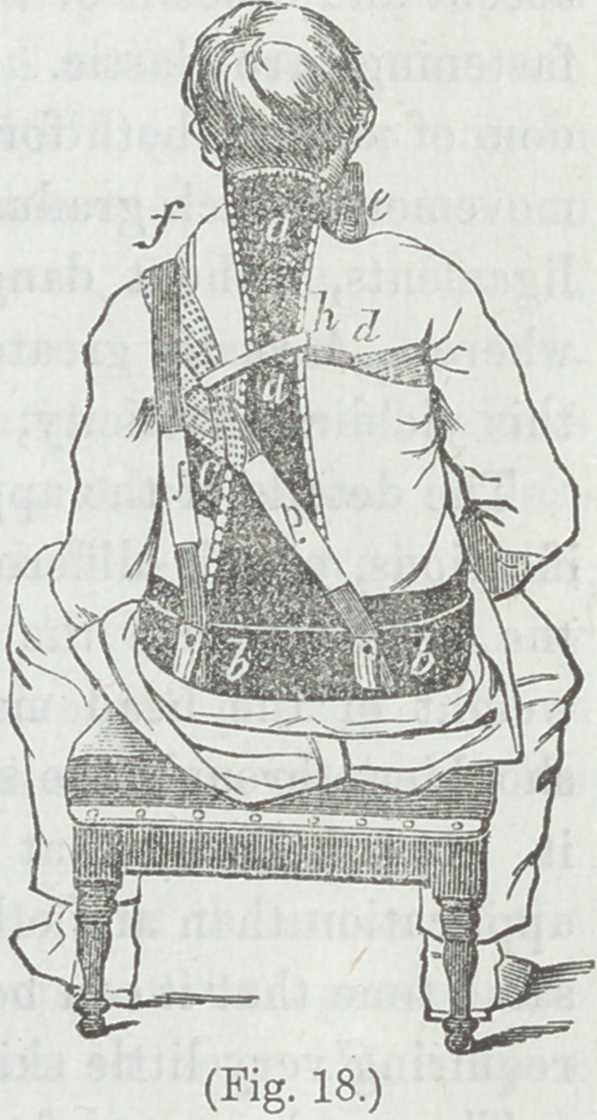


**Fig. 19. f19:**
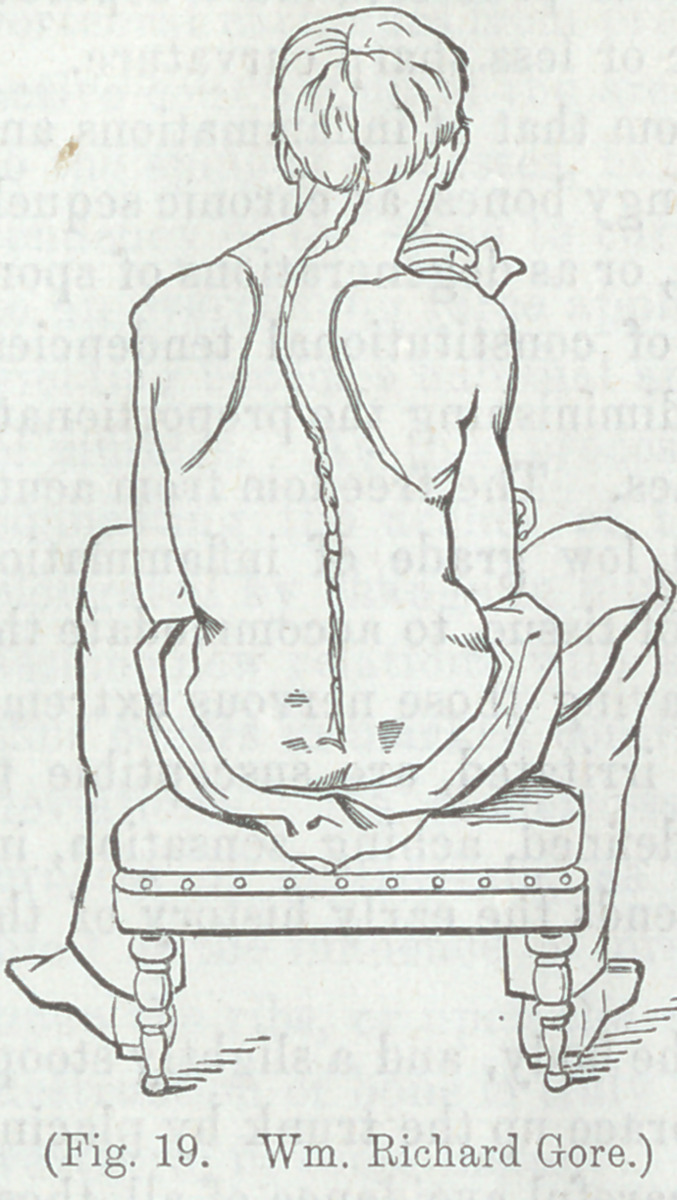


**Fig. 20. f20:**
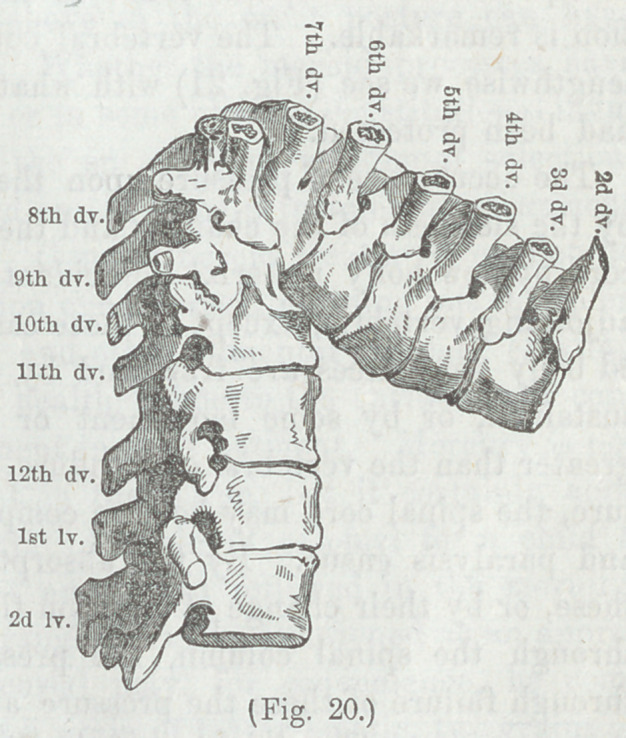


**Fig. 21. f21:**
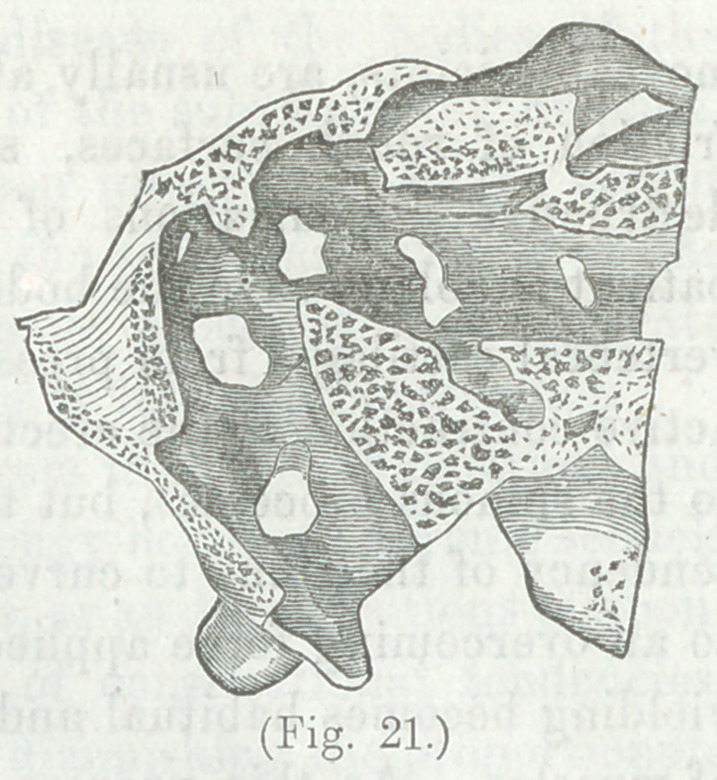


**Fig. 22. f22:**
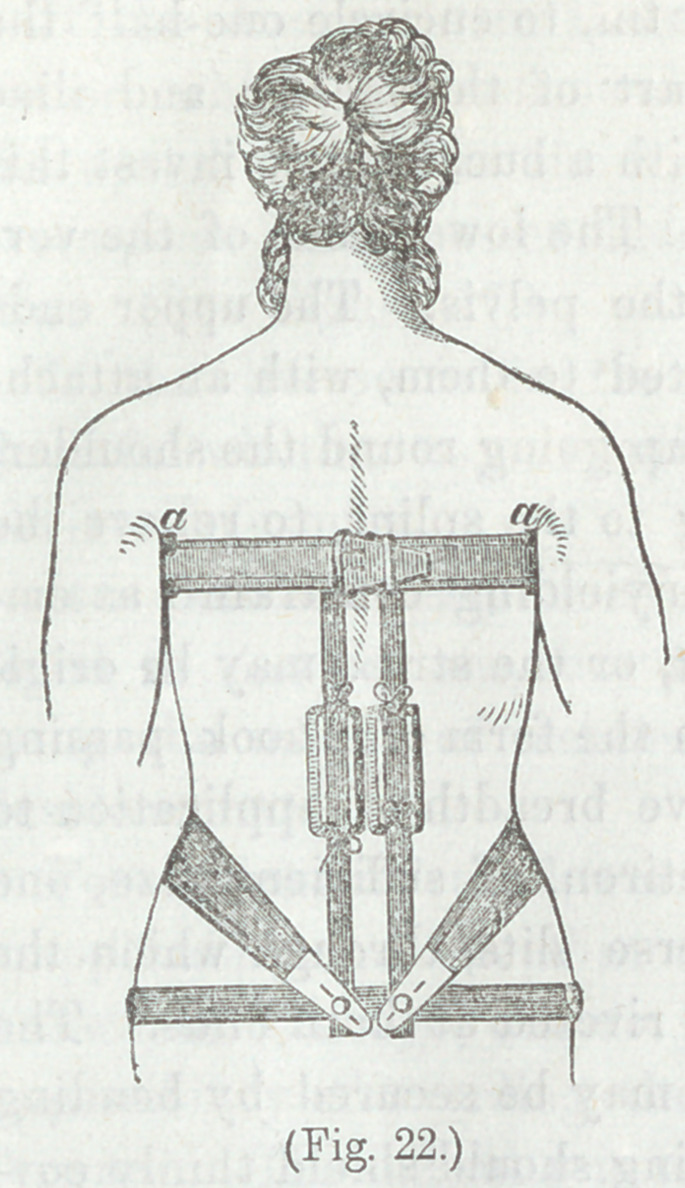


**Fig. 23. f23:**